# In-situ time resolved spectrographic measurement using an additively manufactured metallic micro-fluidic analysis platform

**DOI:** 10.1371/journal.pone.0224492

**Published:** 2019-11-25

**Authors:** T. W. Monaghan, M. J. Harding, S. D. R. Christie, R. J. Friel

**Affiliations:** 1 Ministry of Defence Abbey Wood, Bristol, United Kingdom; 2 School of Chemical and Bioprocess Engineering, University College Dublin, Dublin, Ireland; 3 Department of Chemistry, Loughborough University, Loughborough, United Kingdom; 4 School of Information Technology, Halmstad University, Halmstad, Sweden; University of Sheffield, UNITED KINGDOM

## Abstract

**Introduction:**

Microfluidic reactionware allows small volumes of reagents to be utilized for highly controlled flow chemistry applications. By integrating these microreactors with onboard analytical systems, the devices change from passive ones to active ones, increasing their functionality and usefulness. A pressing application for these active microreactors is the monitoring of reaction progress and intermediaries with respect to time, shedding light on important information about these real-time synthetic processes.

**Objective:**

In this multi-disciplinary study the objective was to utilise advanced digital fabrication to research metallic, active microreactors with integrated fibre optics for reaction progress monitoring of solvent based liquids, incompatible with previously researched polymer devices, in combination with on-board Ultraviolet-visible spectroscopy for real-time reaction monitoring.

**Method:**

A solid-state, metal-based additive manufactured system (Ultrasonic Additive Manufacturing) combined with focussed ion beam milling, that permitted the accurate embedment of delicate sensory elements directly at the point of need within aluminium layers, was researched as a method to create active, metallic, flow reactors with on-board sensing. This outcome was then used to characterise and correctly identify concentrations of UV-active water-soluble B-vitamin nicotinamide and fluorescein. A dilution series was formed from 0.01–1.75 mM; which was pumped through the research device and monitored using UV-vis spectroscopy.

**Results:**

The results uniquely showed the in-situ ion milling of ultrasonically embedded optical fibres resulted in a metallic microfluidic reaction and monitoring device capable of measuring solvent solutions from 18 μM to 18 mM of nicotinamide and fluorescein, in real time. This level of accuracy highlights that the researched device and methods are capable of real-time spectrographic analysis of a range of chemical reactions outside of those possible with polymer devices.

## Introduction

Microfluidics are a technology area that has been applied to specific topics such as: inducing chemical and physical reactions; presenting material samples in a time dependent fashion; performing on device analysis through Lab-on-Chip methods; creating a high sample throughput and continuous replenishment and achieving low sample volumes for high accuracy detection [1,2].

A clear improvement in the capabilities of microfluidics has been directly related to improved production techniques. One of the most significant improvements is the capability to move from very rigid fluidic pathway geometries to much more freeform designs driven by the arrival and development of digital manufacturing techniques, such as Additive Manufacturing (AM) (also known as 3D Printing). Using these advancing technologies, it is possible to forego the rigid multi-stage manufacturing processes required in techniques currently employed and instead produce complex 3D structures in a single stage with minimal user intervention.

This has allowed for expedited development and customization of devices to meet specific needs, reactions, geometries and sample types. Complex mixing, multiple reagent inlets at any location and the ability to integrate with existing hardware are just some of the potential possibilities [3–5]. This unparalleled 3D capacity could allow for true flow synthesis as a result of the potential to facilitate fast fluid flows along with extended residence times. Secondly, adopting an additive manufacturing approach allows the designer/user to perform a more iterative approach to reactor design in which multiple versions of a reactor can be manufactured to dial-in the specific geometries to the specific reaction optimal], with direct feedback coming from the device being redirected back into the design process. This is achieved by forgoing the need to produce a master of replica mould in order to fabricate devices. To date, the AM techniques of material extrusion [5–14], vat polymerization [15–19], material jetting [20–25] and powder-bed fusion [26–29] have been applied to this field. The majority of these already reported devices do however lack one or more of the desired complexity, functionality and resolution and material compatibility required for many modern chemical applications.

In addition to the device geometry and material, in-line and on-line sensing techniques can be used to gain data from substrate usage, product formation, observation of intermediates or the monitoring of reaction parameters such as temperature and pressure on reaction rates [30]. The in-line analysis techniques can avoid issues associated with missing the presence of short lived intermediates which can continue to react (if not quenched appropriately) or decompose, and will therefore not be visible using off-line analysis techniques [31]. It can also be used to overcome issues associated with concentrations and temperature gradients seen in batch reactors, which can reduce the efficacy of vital techniques such as spectroscopic analysis. The majority of common macroscopic chemical analysis systems have achieved miniaturisation due to recent advances in engineering [32,33]. Various detection techniques employed in direct online observation of chemical and physical events include fluorescence [34] and optical absorption spectroscopic methods (ultraviolet-visible and infra-red) [35] and nuclear magnetic resonance (NMR) techniques [36].

The combination of ‘in-line’ measurement, small sample volumes, and continuous monitoring in a more robust microfluidic device would allow for a large range of sample materials and reagents to be investigated in high detail. This investigation could be performed at elevated temperatures and pressures with techniques that are not favourable to standard AM materials and techniques (e.g. high UV, hard and soft X-rays and neutron sources).

**Fig 1:**
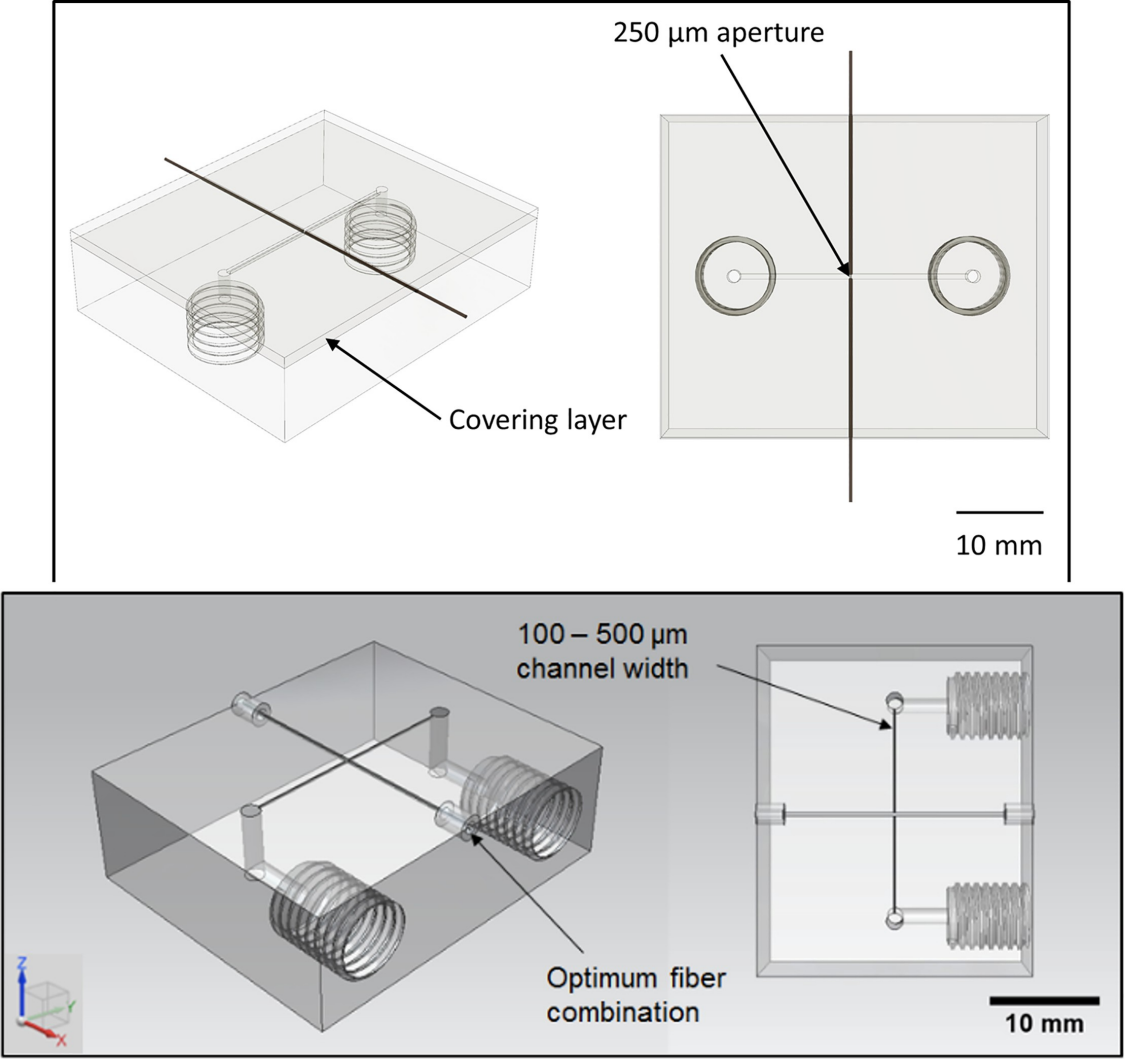
Schematic of the first reported example of a UV-vis UAM reactor cell [4].

One promising technique that has yet to be explored in the manufacture of microfluidic devices is Ultrasonic Additive Manufacturing (UAM), which comes under the AM class of sheet lamination technologies. UAM is a hybrid, bond-then-form sheet lamination technique that combines ultrasonic metal welding (USW) and computer numerical controlled (CNC) machining to create complex metal parts with computer aided design defined shapes and features [37]. The USW process differs substantially from other welding processes in that ultrasonic welding is a solid-state process in which bonding between foils occurs at temperatures < 50% of the melting point of the material to be consolidated [38–40]. Welding between foils occurs autogenously (i.e. they are self-fused, without the addition of solder or the application of an adhesive). An overview of the process is located in Fig 1 for the manufacture of a simple hypothetical flow reactor.

UAM has the potential to form highly robust and complex metallic devices suitable for performing high-temperature and high-pressure chemistry. UAM has the ability to develop multi-material structures including, potentially, catalytic materials such as Copper (Cu) and Iron (Fe) [41–43]; embedded components such as various fibre types [3,44–48] and electronics [49–52]; the hybrid nature (material deposition followed by selective removal) and non-powder based nature of this metal-based AM technique have substantial future implications for the formation of highly complex 3D reactors.

The authors have previously made an advance in creating UAM parts with embedded spectroscopy ability. This paper presents the first in-situ, small separation gap UV-vis spectrographic time resolved scientific study of microfluidic chemical reactions using a metal based AM technique.

## Materials and methods

### Microfluidic device

To demonstrate the accuracy of the in-situ spectrographic analysis capable of being delivered, a μ-total analytical system (μ-TAS) was manufactured. Due to the advantages afforded by AM in the development of reactor systems, the test device was designed virtually and then manufactured direct from the CAD data. The layout of the device structure was chosen to establish how effective/ineffective the embedded optical fibres were at transmitting and collecting light across an aperture of fixed width (250μm, Fig 1); and is similar to what has been reported in previous publication [53].

The microfluidic device was created using a combination advanced production technologies: Ultrasonic Additive Manufacturing (UAM) to create the fluidic device body and embed the optical fibres; electrochemical etching to form fluidic channels and access the embedded fibres; and Dual Beam Focussed Ion Beam (DBFIB) to cut the measurement space in the embedded optical fibres with in-situ polishing of the optical surfaces. The production specifics for this device are covered in the supplementary material–S1 File.

Previous work by the authors has established the ability to directly embed metal-coated optical fibers within the interlaminar regions of UAM substrates, whilst maintaining full functionality [3]. Additional authored work has also shown that spectroscopic sampling can be achieved with high degrees of success when path lengths of 100–500μm are used [4]. It was also shown that reduced path lengths lead to increased working concentration ranges. Whilst the width of this channel will not directly affect the path length used in later spectroscopy, it is desirable to keep the channel width consistent and within the typical sub-millimetre range; this is to prevent changes in flow rate as a result of increased hydraulic diameter whilst retaining the advantages associated with reduced channel sizes.

### Materials

For the microfluidic device body a particularly corrosion resistant Al-Mn alloy was chosen, Al 3003 H18. The reasons for this choice were: high corrosion resistance suits the application to more aggressive sample materials/reagents [54], the behaviour of Al 3003 (H18) under UAM processing conditions is well understood [55,56] and it has been proven to be compatible with the embedment of several types of fibre, including optical fibres [44,47].

The chosen optical fibres integrated into the microfluidic device were aluminium-coated graded-index, multimode optical fibres with a 100-μm silica core. The dimensions and compositions of these fibres are located in Table A in S1 File. The choice of diameter and multimode fibres was empirically determined based on increasing light transmission and correct alignment facilitation.

### Validation of embedded optical fibre transmission

After the microfluidic device production the transmission performance of the embedded optical fibres was established through spectroscopic transmission analysis. This was to ensure suitable performance for later spectroscopic measurements in which analyte detection and quantification were investigated.

For qualitative analysis of the effect of DBFIB cross-sectioning the embedded fibres, the embedded optical fibres were connected to a Deuterium-Halogen light source (DH-2000, 215-2500nm, Ocean Optics, Oxford, UK) and an S2000 UV/Vis spectrometer with a USB1000-ADC analogue to digital converter (Ocean Optics, Oxford, UK) via the use of bare fibre adapters (multi-mode SMA connector, Newport Spectra-Physics Ltd, Oxford, UK). A measure of the light transmitted across the newly formed aperture was then recorded at wavelengths of 260 and 494 nm for various time intervals to establish the degree of light transmitted through the fibres. These wavelengths were chosen specifically to relate to later work on in-line analyte detection. The cell was first filled with de-ionised water in order to collimate transmitted light and provide a more accurate representation of the working cell. The spectra produced were then compared to the transmission performance of embedded, non-milled optical fibres to establish any effect of UAM embedding on fibre functionality

It was established that DBFIB milling was a suitable method for precise fibre cross-sectioning whilst retaining full optical properties. An investigation into the spectroscopic performance of UV-Vis flow cells featuring these fibres was performed to establish the upper and lower limits of detection of the cell as well as assessing the potential for this technology in future applications. This separation distance of 250 μm is comparable to path lengths used in previous works regarding on-chip UV-vis detection and provides a compromise between the extended distances usually required for low concentration samples and the short distances required for high concentrations.

### Analyte detection and quantification

The validated UAM microfluidic device was used to analyse two different aqueous solutions containing different organic compounds. As with the validation method, the devices optical fibres were connected to a Deuterium-Halogen light source and a UV/Vis spectrometer via the use of bare fibre adapters. A measure of light absorbance across an aqueous solution filled channel was then recorded at 260 and 494 nm (corresponding to the lambda max of the selected compounds) at fixed time intervals to establish the degree of absorbance at set concentrations.

To firstly test the upper working concentration limit of the device, solutions of the UV-active water-soluble B-vitamin nicotinamide (Acros, 99% purity) was chosen. This compound was chosen as it has a relatively low value of molar absorptivity and it is therefore likely that this technology will be applicable to a wide range of organic compounds if successful in these more challenging measurements. Using a value of 2650 LM^-1^ cm^-1^ for the molar absorptivity, ε, [NIST Standard Reference Database, No. 69] the appropriate concentration range to test the cells was determined by the Beer-Lambert law (Eq 1) [57].

$$A_{abs}=\frac{I_{0}}{I}=\varepsilon cL$$ (1)

Aabs = Absorbance (a.u)

Io = Incident light intensity

I = Transmitted light intensity

ε = Molar absorptivity (L mol^-1^ cm^-1^)

c = Sample concentration (mol L^-1^)

L = Path length (cm)

Solutions were prepared from 1 to 30 mM in order to provide a thorough working range (Table 1). The upper detection limit of the cell is of equal importance to the lower detection limit for flow chemistry where maximal throughput is achieved by using as high a working concentration as possible with the constraint of minimising solvent consumption. The higher concentration nicotinamide solutions should therefore identify the linear dynamic range for this system and establish an estimate for the concentrations that could potentially be employed in both reaction monitoring and optimisation.

**Table 1:** Nicotinamide concentrations for microfluidic flow cell testing.

Solution	Concentration [mM]
1	1.00
2	2.50
3	5.00
4	7.50
5	10.0
6	12.5
7	15.0
8	20.0
9	25.0
10	30.0

The acquisition time was then adjusted so that approximately 3000 counts were recorded by the spectrometer at the selected wavelengths. This number of counts is specific to the S2000 UV/Vis spectrometer and ensured that a sufficient intensity of light was recorded by the detector without any saturation occurring at the detector. A total of 10 averaged spectra were used for each measurement and all spectra were recorded with 3 point boxcar averaging. The prepared solutions were then pumped into the device, starting with the lowest concentration, and the absorbance value measured prior to rinsing with water between each solution to ensure minimal carryover.

In regards to analyte detection, it is also desirable to be able to work at the lower end of the concentration range where the emergence of small quantities of side products etc. is likely to appear. Therefore in order to estimate the lowest concentration that could be analysed using these cells, a compound with a high molar attenuation coefficient was utilised to determine the cells limit of detection (LOD). The LOD is commonly defined as the lowest quantity of a substance that can be distinguished from the absence of said substance. The compound chosen for these measurements was fluorescein, a synthetic water-soluble organic compound with a molar attenuation coefficient of 76900 L mol^-1^ cm^-1^. A dilution series was then formed from 0.01–1.75 mM and the LOD for each cell determined using Eq 2 [58];

$$LOD=regression\;intercept+3S_{B}$$ (2)

$$s_{B}=\sqrt{\sum_{i}\frac{{\left(y_{i}-{\hat{y}}_{i}\right)}^{2}}{n-2}}$$

Where;

y_i_ = absorbance value (a.u)

ŷ_i_ = ‘fitted’ absorbance value (a.u)

n = number of samples

S_B_ = Standard deviation of a blank sample

The regression intercept was the value for c for the generated y = mx + c equation, when x = 0 as it was based on a univariate calibration.

A dilution series was formed from 0.01–1.50 mM and the LOD for each cell determined using Eq 2 [58]. The exact concentrations used for the dilution series of Fluorescein are located in Table 2.

**Table 2:** Dilution series used for LOD determination using fluorescein.

Solution	Concentration [mM]
1	0.010
2	0.020
3	0.041
4	0.081
5	0.163
6	0.325
7	0.750
8	1.000
9	1.500

## Results and discussion

### Device manufacture

**Fig 2:**
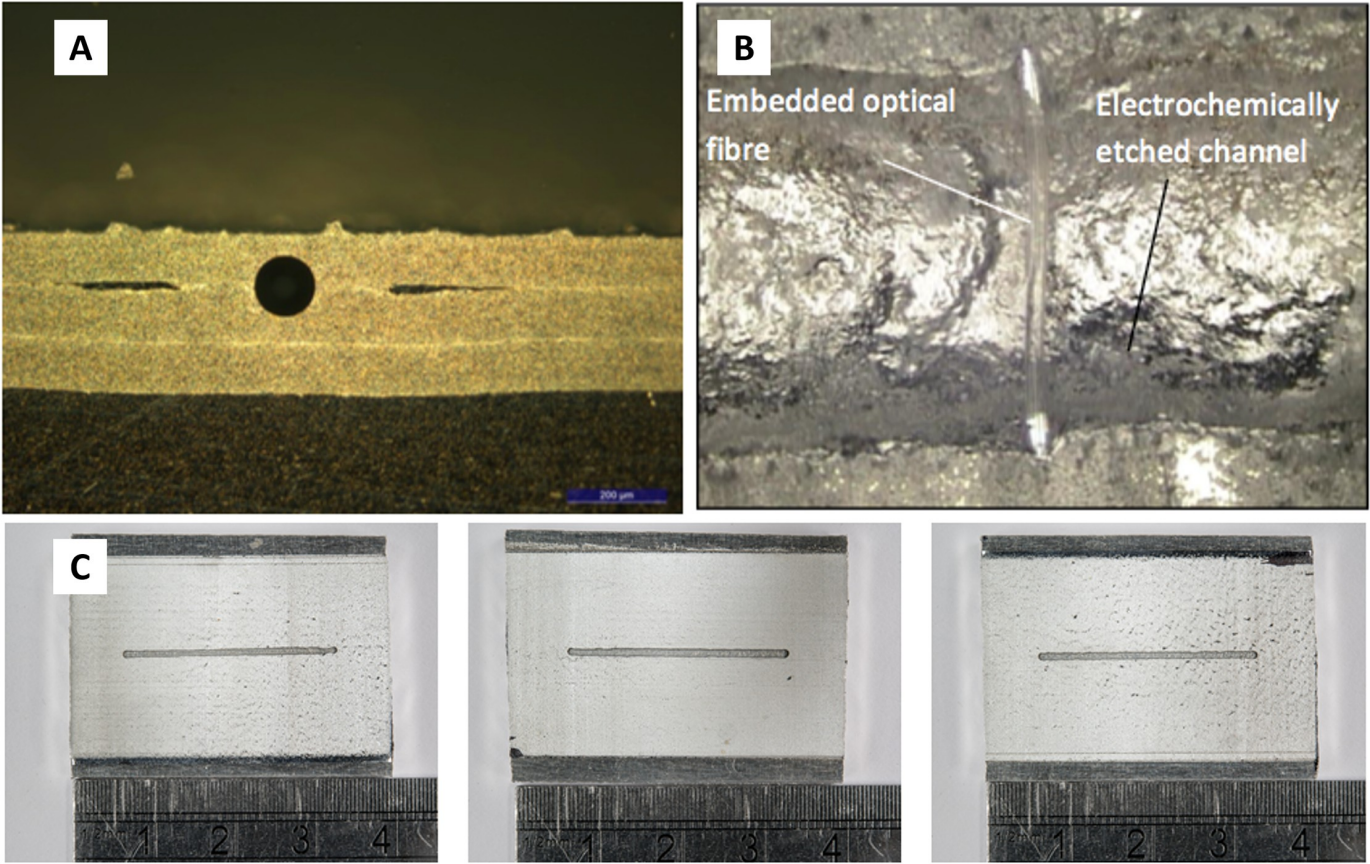
a) image of aluminium-coated optical fiber embedded via UAM b) exposed optical fibre exposed via electrochemical etching c) refined electrochemical etching results.

Following methods laid out in previous publications [3], aluminium-coated optical fibers were embedded within the interlaminar region of the selected Al 3003 foils (Fig 2A).

Due to the fragile nature of optical fibres when subjected to methods such as CNC milling, a means of forming fluidic channels around these embedded fibres needed to be established in order for them to be cross-sectioned at a later stage. As a result, electrochemical etching was selected as a test method for producing fluidic pathways around embedded features (Fig 2B). Systematic experimentation in which variables were altered established that the channel formed at 625 mA and 2.5 minutes demonstrated the lowest degree of under etching (ca. 100μm) and was also able to suitably expose the embedded optical fiber (Fig 2C). The degree of under etching was the difference between the etched channel width and the width of the channel in the resist applied to the surface of the UAM substrate and was quantified using the micrograph analysis.

Additional images and results from the full range of parameters tested can be found in the supplementary materials–S1 File.

**Fig 3:**
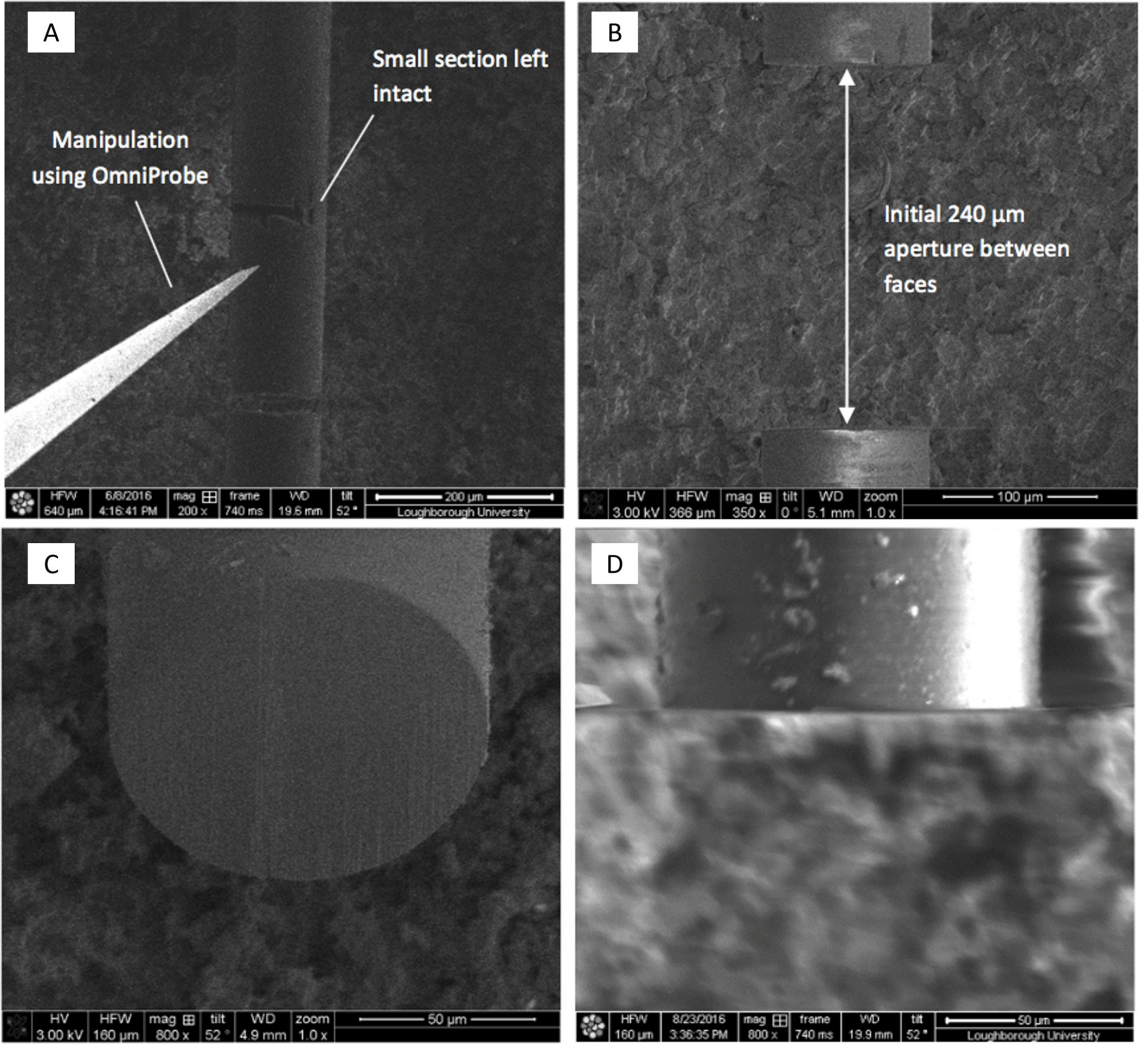
a) manipulation of embedded fiber using OmniProbe coaxial needle b) aperture produced via fibre manipulation at initial 240-μm distance c) and d) optical surfaces post ion-beam polishing.

Through the use of ion beam milling, a method of cross-sectioning embedded optical fibres was developed and found to be highly suitable in the formation of optical configurations applicable to UV- Vis spectroscopy, The fibre was initially cross-sectioned using the ion beam to mill away small rectangular segments, from one side of the fibre to the other (Fig 3A). Due to issues with the resultant highly charged surfaces occasionally causing milled away sections to stick to the remaining bulk material, a small section of the fibre was left intact in order to allow manual removal of this cross-section.

Using the DBFIB coaxial OmniProbe needle to manipulate this cross-section segment, the segment was removed to create a face-to-face aperture of 240 μm (Fig 3B). The faces of these fibres were course and required additional polishing in order to produce both clean flat faces and adjust the separation distance to the desired 250 μm +/- 10 (Fig 3). Using a reduced aperture, these faces were highly polished to yield clean, flat surfaces that are highly desirable for the transmission and receiving of light across a band gap (Fig 3C and 3D). In spectroscopy, this reduces coupling losses increasing detection sensitivity, however the flat parallel surfaces with the fluid fill cavity in between could have induced unexpected variations in transmissivity and reflectivity as a function of the wavelength. It was noted during measurement work by the authors that the acquired absorbance spectra were visually good and attenuation and reinforcement of incident light were expected to be normalized, based on the assumption that the reference spectrum would include similar effects which did not vary from measurement to measurement. Spectral distortion at specific wavelengths were not noted suggesting that Fabry Perot effects were not being encountered during measurement the work. This suggests that any unexpected variations were mitigated and/or not present within the manufactured device.

Concluding this final FIB polishing stage a covering Al 3003 H18 layer was used to seal the etched fluidic pathway. Once embedded, the system was taken forward for spectroscopic testing in regards to the transmission performance of the cross-sectioned fibres.

### Embedded optical fibre transmission performance

**Fig 4:**
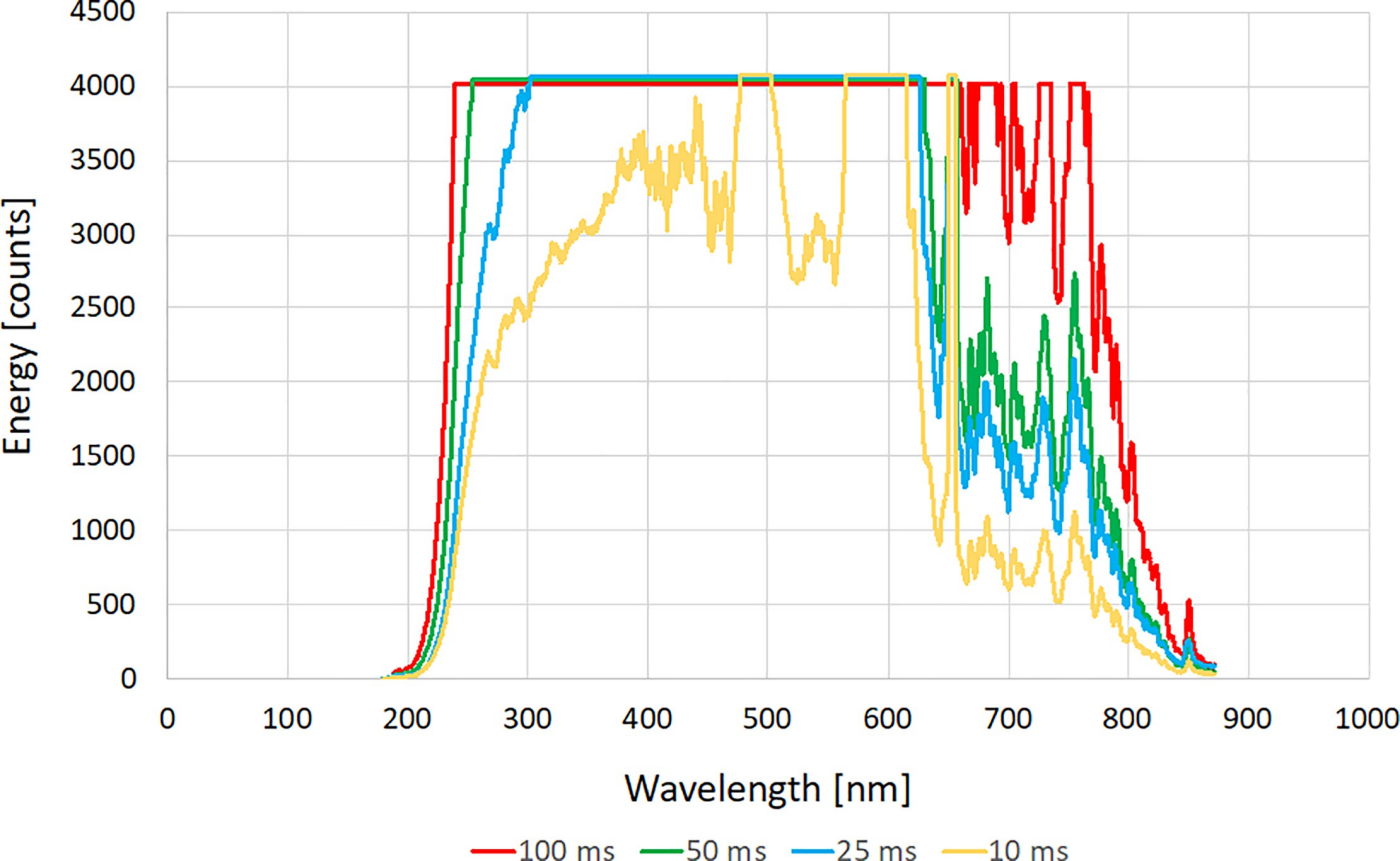
Transmission spectra recorded for UAM UV-vis cell at varying acquisitions times (10–100 ms).

After device production the transmission performance of the embedded optical elements and aperture combination were tested. This was done by determining the degree of light transmission across a de-ionised water filled channel at various wavelengths and acquisition times. The transmission spectra obtained from the reactor cell with acquisition times from 10 to 100 ms are located in Fig 4. The acquired spectra match the expected line shape for the deuterium light source with the characteristic deuterium emission bands at ~ 500, 600 and 650 nm shown in Fig 4.

This testing revealed that even with short acquisition times high degrees of light transmission were recorded across the aperture by the opposing detector. This high degree of light transmission can allow the user to adjust the system by reducing the acquisition time so that the detector is not saturated at the wavelength of choice, as is seen in the case of the 10ms acquisition. The ability to gather clear spectroscopic data over short acquisition times is ideal for those looking to both monitor reactions online for optimization purposes and those looking to establish the emergence of short-lived intermediaries or side products.

Conclusively, this method has demonstrated that FIB micro-fabrication is highly suited for the development of accurate optical arrays embedded within UAM substrates. The precise site selection, milling control, and high-quality surface finish enable this fabrication technique despite the confines of the fibre within the micro-channel. This technique has the potential to afford direct monitoring via *in-situ* integration where spectroscopic measurements are focused directly inside the micro-channel [103]–[105]. This direct monitoring approach usually comes with a reduction in portability as a result of large optical set ups. The functionality of these devices for such monitoring applications was established through the manufacture of a UV-vis flow cell combining UAM technology and the electrochemical etching cell-DBFIB methodology, marking the first published work of its kind.

### Analyte detection and quantification

**Fig 5:**
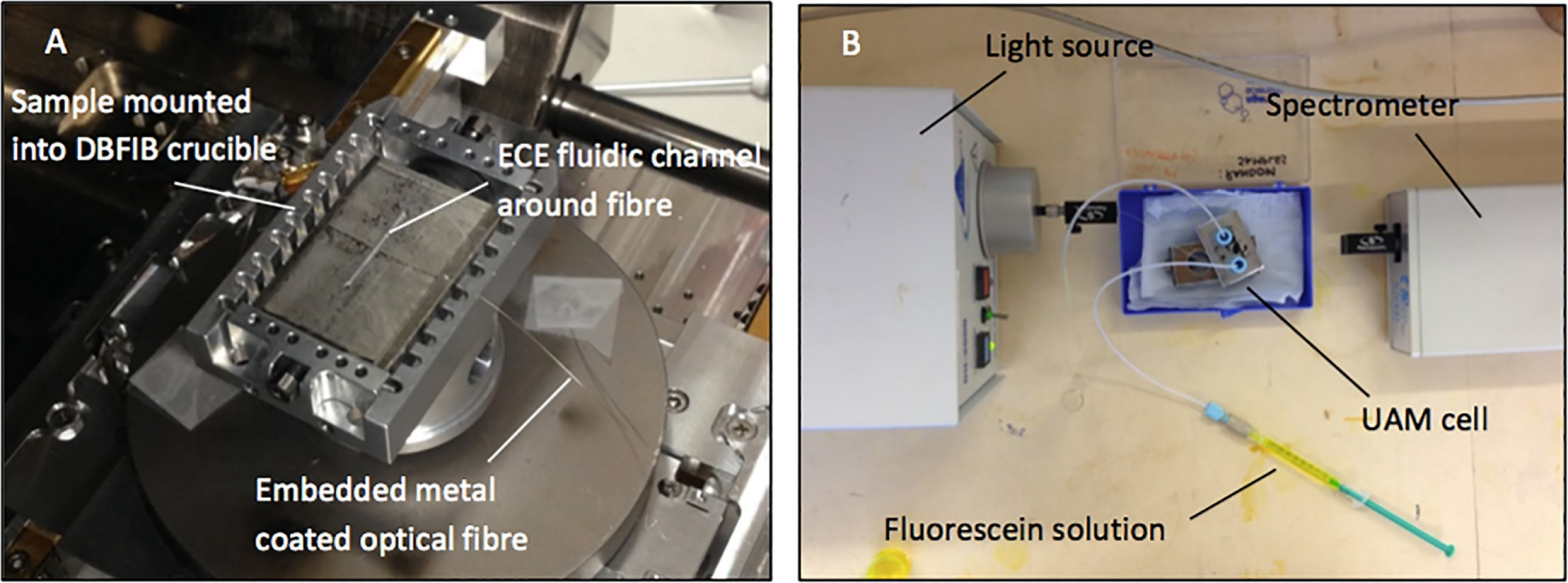
(A) UAM cell featuring embedded fibre mounted for DBFIB work (B) configuration used in analysis of nicotinamide and fluorescein solutions.

Nicotinamide solutions were used to measure the upper working concentration range of organic compounds possessing middling molar attenuation coefficients when using these devices, whilst Fluorescein was used to establish the lower end of detectible concentrations. The calibration curves produced using this compound were then used to estimate the LOD of these cells. Fig 5 displays the machined cell equipped with embedded fibres being mounted for DBFIB milling and the set-up used with the finished cell.

**Fig 6:**
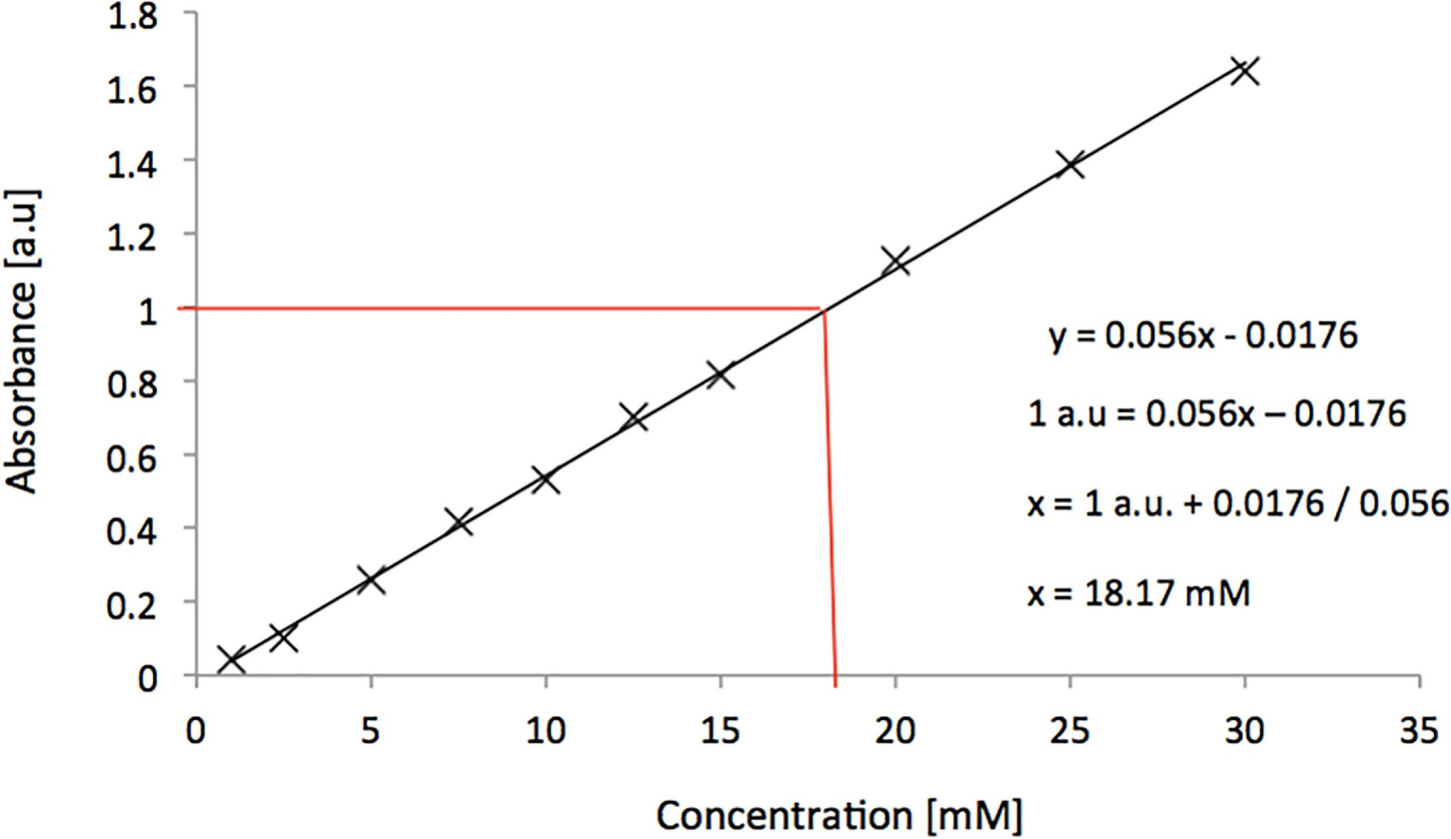
Linear absorbance calibration curve obtained using UAM cell with nicotinamide solutions of various concentrations.

**Fig 7:**
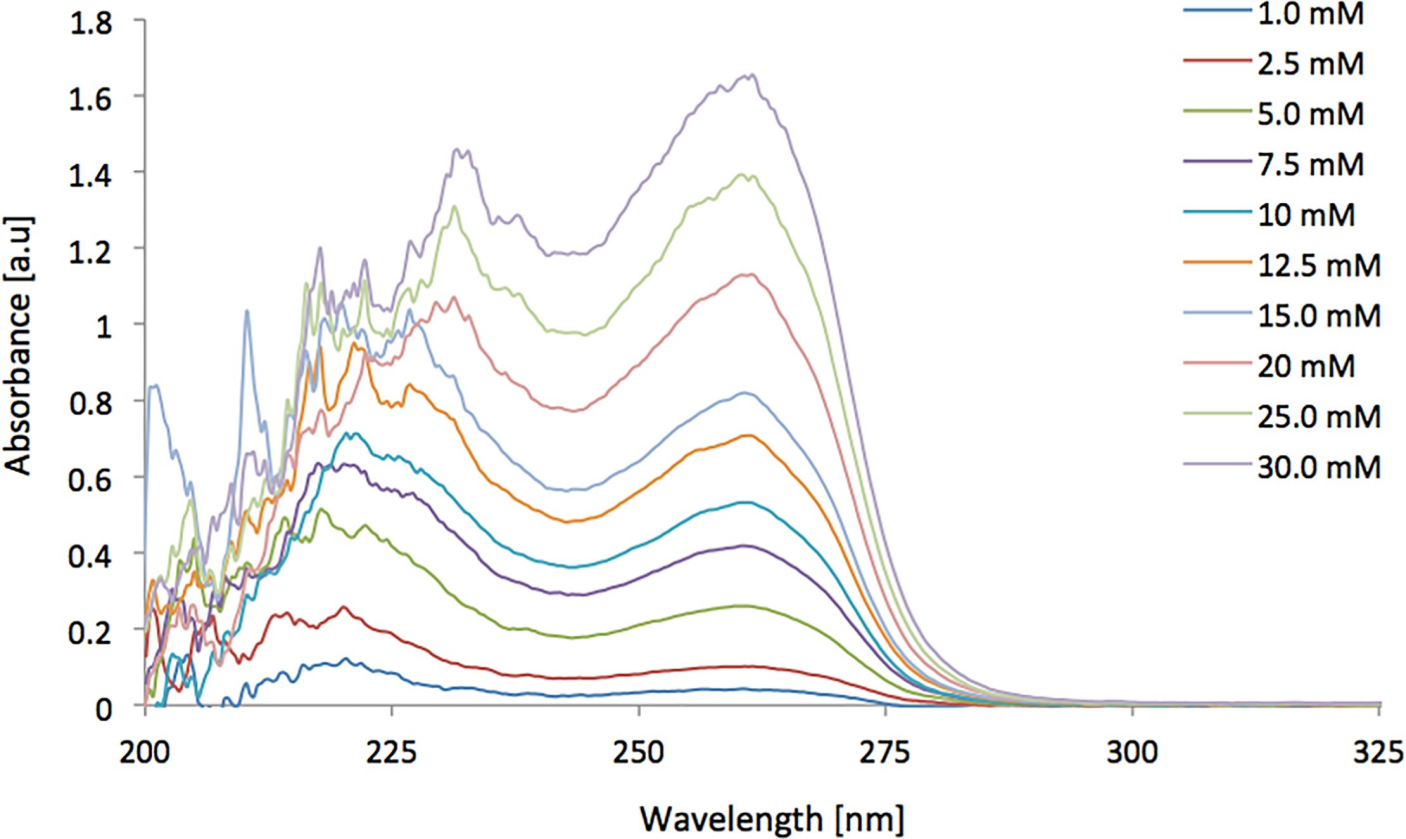
Nicotinamide absorbance spectra generated in the UAM cell featuring a path length of 250 μm.

Linear regression analysis for the measured absorbance against known concentration values for the cell was highly linear (R^2^ > 0.999) across the entire range of solutions (Table 1). This range of concentrations is well suited for online flow chemistry analysis as higher working concentrations facilitate increased throughput. This wide of a range of concentrations is usually not possible when employing a standard commercial UV-Vis flow cell as a result of the significantly larger light transmission path lengths (1-10mm). These larger path lengths result in stronger absorbance measurements than would have been seen in their shorter path lengths for the same concentration. Despite these linear responses, absorbance readings significantly greater than 1 a.u are generally not considered reliable for quantitative measurements. When absorbance is > 1.0 a.u, approximately 90% of the transmitted light is being absorbed by the solution leaving approximately 10% to be collected by the opposing optical fibre. Therefore, even a small error in light detection can cause a large error in the analysis. Due to this, it is usually advisable to work within an intermediate range of absorbance readings of 0.2–1 a.u. As a result, in this cell, the maximum theoretical concentration that could be handled reliably is approximately 18 mM, based on regression analysis with a fixed absorbance of 1 a.u (see Fig 6). The absorbance spectra obtained for the nicotinamide solutions at the various selected concentrations are shown in Fig 7.

Previous work conducted in parts produced using a vat-polymersation technique for devices of similar layout were shown to produce an upper working concentration limit of 15 mM for 200 μm path lengths and 12.5 mM for 300 μm path lengths. The increase in working concentration was attributed to the highly polished faces and accurate fibre placement allowing more efficient fibre coupling. The capability of the device is as a direct result of the combination of UAM with channel etching and DBFIB machining.

**Fig 8:**
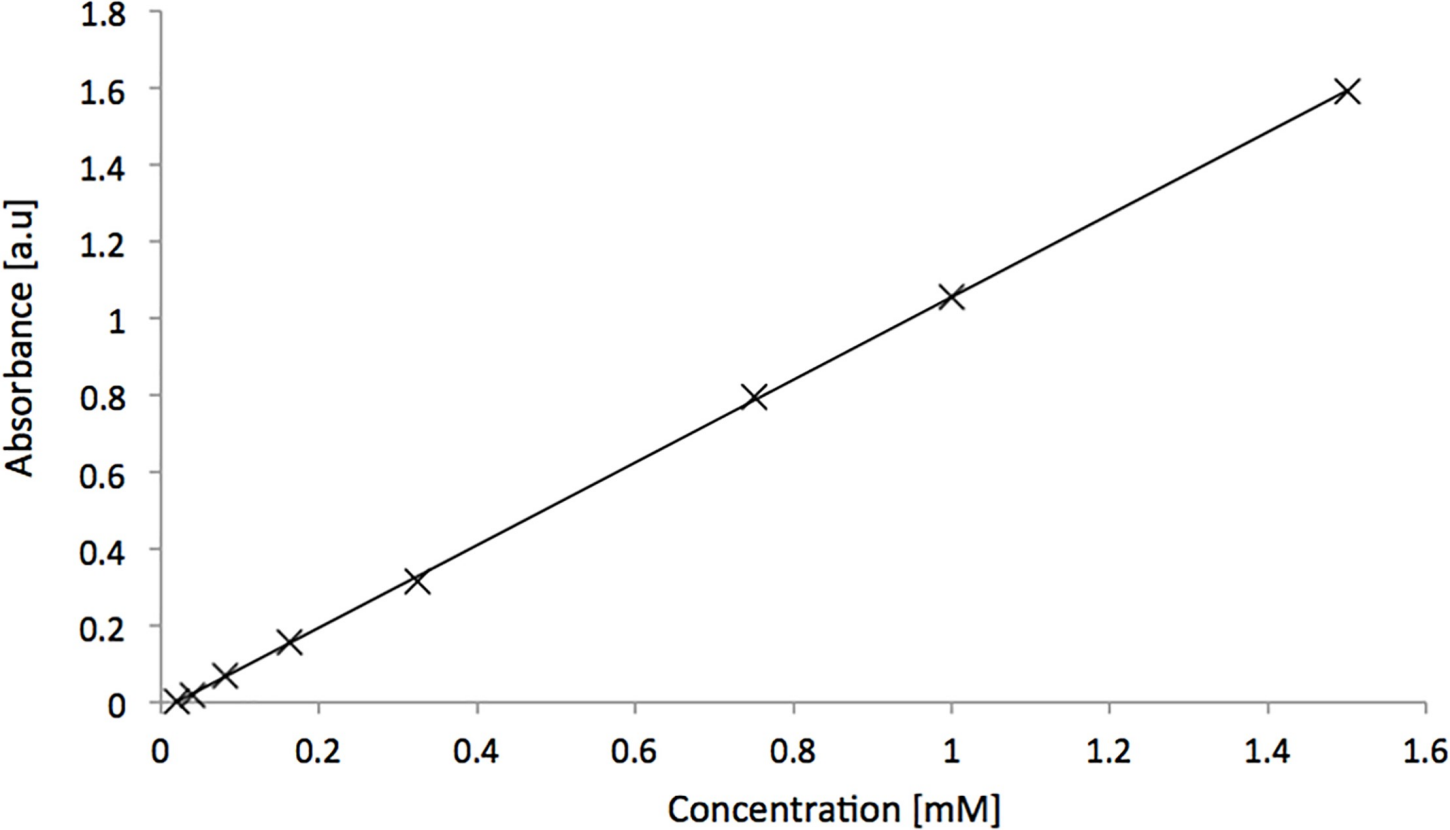
Linear absorbance calibration curve obtained using UAM cell with fluorescein solutions of various concentrations.

**Fig 9:**
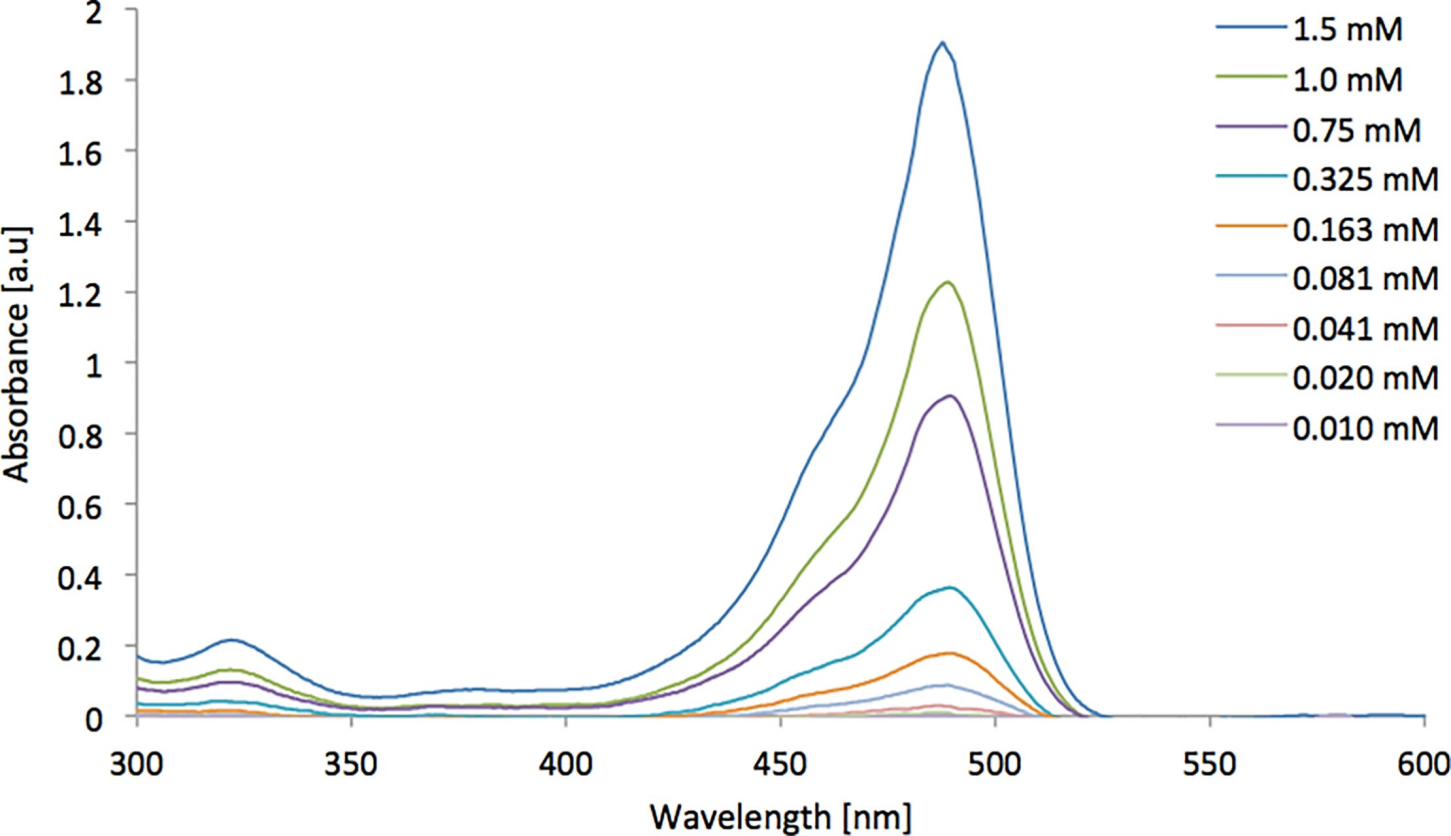
Fluorescein absorbance spectra generated for the UAM cell featuring a 250 μm path length.

As well as establishing the working range for potential use in in-line reaction monitoring at high concentrations, the LOD for the cell was also established using a compound with significantly higher molar attenuation coefficient, in this case an aqueous dilution series of fluorescein. These data were then used to establish the theoretical limit for qualitative analyte detection within this system. Fig 8 shows the liner absorbance calibration curve for these fluorescein solutions whilst Fig 9 shows the different absorbance spectra obtained for the dilution series. The corresponding calibration curve was then used to estimate the cells LOD.

Through the generation of this data, the theoretical limit of detection for the cell could be calculated from Eq 2. This calculation yielded a limit of detection for the system of 18 μM, meaning that the cell is capable of measuring solutions from 18 μM to 18 mM. This wide range of concentrations is a significant advantage for online reaction monitoring in which high throughput is attained when using as high a concentration as possible whilst the high degree of system sensitivity is useful for low-level analyte detection.

This direct incorporation of these optical elements has the potential to transform UAM devices into powerful analytical tools for determining optimal reaction conditions, the presence of analyse and reaction kinetics. In these UAM systems however, there are no issues associated with organic solvent compatibility or elevated temperatures and pressures. This enables a wide range of organic reactions that could potentially be performed and monitored within the system, providing they possess suitable UV-activity. This work demonstrates that UAM permits the use of metallic materials with fluidic pathways and detector capabilities on par of traditionally manufactured polymer devices. The improved compatibilities are further expanded by the more robust nature of the fluidic device material and the greater fluidic path freedom that is possible via an AM approach. This work has resulted in highly robust devices capable of facilitating wide ranging reaction conditions and multiple potential further applications such as UV catalysis and chemiluminescence detection.

## Conclusions

This work has demonstrated how the application of advanced production technologies to the field of microfluidic reactionware can result in new possibilities for the field in terms of more robust devices applicable to a wider range of reactions whilst maintaining measurement accuracy in a more freeform design. In addition the fact that the tested organic solvents do not affect the device materials (i.e. Al) in this work means the lifetime and range of applications is superior to previously demonstrated AM polymeric parts.

The precise nature of DBFIB milling and lift out procedures means that very high-resolution optical configurations can be formed. The highly polished nature of the fibres produced in combination with their accurate alignment leads to excellent fibre coupling, increasing the light transmission for improved analyte detection

## Supporting information

S1 File Microfluidic device manufacture. Details of the process chain, the manufacturing techniques and the parameters used to manufacture the microfluidic devices used in this research paper.
(PDF)
