# Supplementary material for: In-situ time resolved spectrographic measurement using an additively manufactured metallic micro-fluidic analysis platform
Source: PLoS One. 2019 Nov 25;14(11):e0224492. doi: 10.1371/journal.pone.0224492 (PMC6876875; doi:10.1371/journal.pone.0224492)
Supplement: S1 File — Details of the process chain, the manufacturing techniques and the parameters used to manufacture the microfluidic devices used in this research paper. (PDF) [file pone.0224492.s001.pdf]

Equipment

All UAM samples detailed in this work were prepared using an Alpha 2 UAM machine (Solidica Inc., Ann Arbor, Michigan, USA) located within Loughborough University's Mechanical and Manufacturing Engineering department.

A Nova 600 NanoLab, ultra-high resolution (UHR), field emission gun (FEG) – SEM/focussed ion beam (FIB) machine (FEI Company, Oregon, USA) was employed for both sample cross-sectioning and imaging. This apparatus delivers a focused Ga^{+} ion beam with energy 5–30 keV and a probe current of 1 pA - 20 nA.

Prior studies conducted by the authors of this paper have established the ability to embed optical fibres at UAM parameters conducive to the formation of high strength metal matrix composite structures, with little to no damage occurring to the fibres, as a result of protective metal jackets applied to their circumference [1]. This work was conducted in order to provide the foundation for future spectroscopic interrogation directly within the fluidic channels of UAM devices such as those presented in other prior works [2]. This was done with the intention of achieving the advantageous properties associated with in-line reaction monitoring and additive manufacturing.

Preparation of UAM samples

The process chain of sample fabrication is demonstrated in Fig A. The samples to be taken forward into etching were prepared by first depositing five separate Al 3003 H18 foil layers (100μm thick) onto an Al 1050 supporting plate, at 21 $\pm$ 5°C, to form a UAM metal matrix.

The parameters used for the deposition of these foils were 1400N normal force, 20µm oscillation amplitude and a 40mm/s welding speed. These parameters were obtained via systematic tests and prior studies focusing on the ultrasonic embedding of metal-coated optical fibres in Al 3003 H18 metal matrices [1].

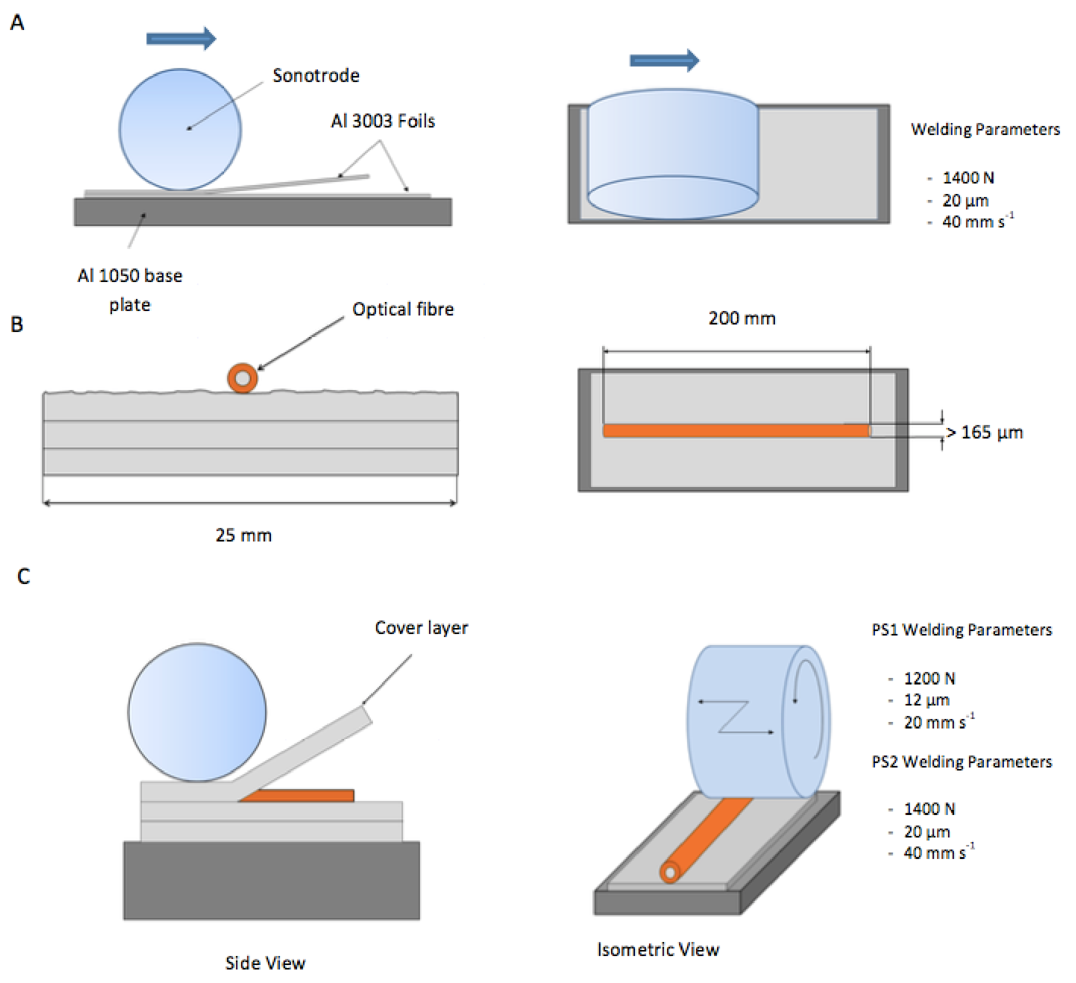

Fig A. Process chain of fabrication. (A) substrate manufacture (B) optical fibre placement (C) encapsulation of fibre. Note the covering layer is not displayed in the isometric view of (C) for the purpose of clarity

200 mm lengths of the selected Al-coated optical fibre were placed onto the surface of the substrate, along the direction of consolidation, and fastened in place using temporary fixturing.

This temporary fixture was required to stop the fibre moving significantly during the welding process as was noted in preliminary trial runs.

Electrochemical cell manufacture and initial exposure testing

Due to the fragile nature of optical fibres when subjected to methods such as CNC milling, a means of forming fluidic channels around these embedded fibres needed to be established in order for them to be cross-sectioned at a later stage. As a result, electrochemical etching was selected as a test method for producing fluidic pathways around embedded features.

This process begins by first etching the narrowest channel possible into the surface of the polymer etch resist applied to the surface of the UAM substrates. This value was determined to be ca. 550 μm and was achieved using a combination of 20% laser power and 1100 mm/s scanning speed. Using these parameters, multiple substrates were produced and taken forward into testing to establish the effects of a various parameters in an ECE cell on the width and depth of channels produced.

A DOE approach was taken to the formation of fluidic channels onto the surface of the UAM substrates utilizing the electrochemical etching cell in order to establish the most suitable range of parameters to be used in later optimisations. The responses examined from these different combinations of parameters centred on both the width and depth of the produced channel. This was achieved through non-contact focus variation microscopy using an Alicona surface profiling system. The desired result of these trials was to produce a channel as close to the width of the overlying aperture in the tape as possible through reducing the effects of under-etching

and producing a depth whereby the core of a silica fibre could be exposed (ca. 200 µm) for later work to fibre cross-sectioning. The results of these initial DOE trials are displayed graphically as response contour plots within the appendix.

The sample producing the cleanest channel with the narrowest diameter and appropriate depth (> 200 µm) was the sample with parameter combinations of 750mA and an exposure time of 5 minutes. This combination produced a channel depth of 375 µm and a width of 841 µm. As a result, an optimisation DOE was performed, with this parameter combination acting as the design space central point. Both time and current increments were equally spaced either side of this centre point in order to try to optimise the etching result. As with previous trials, experimental runs were both randomised and repeated. The parameters used for this are located below in Table 1.

Table A. Parameters used in etching optimisation DOE

Factor	Low	Centre	High
Exposure time [min.]	2.5	5	7.5
Current [mA]	625	750	875

Samples identical to those used in previous trials were formed and used in this optimisation and again measured in order to determine the response in width and depth of the channels formed because of changes in current and exposure time. The resulting response contour plots for the responses within the range of samples are located the appendix.

In contrast to earlier trials, the channels formed with the lowest exposure time (2.5 min) at each of the different current settings (625, 750 and 875 mA) were seen to exhibit consistent channels

with clean edges and consistent depths. Images of these optimised samples are located in Fig B whilst the resulting measurements are displayed in Table 2.

taken forward for etching trials.

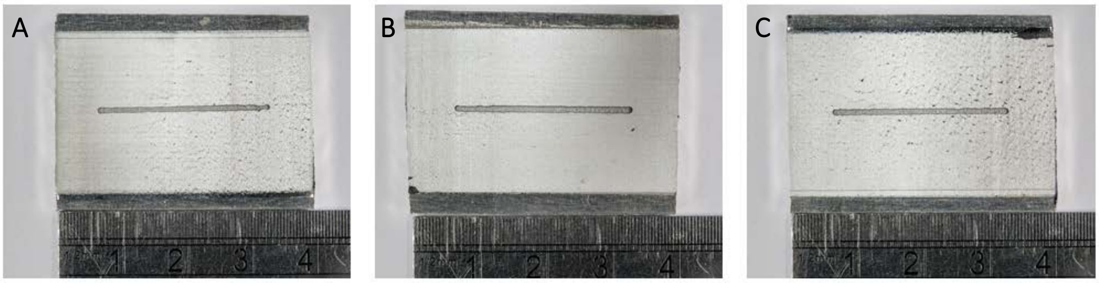

Fig B. Optimised samples at (A) 625 mA and 2.5 min (B) 750 mA and 2.5 min and (C) 875 mA and 2.5 min

Table B. Profile responses from optimised parameter ranges

Optimized parameters	Average width [μm]	Average depth [μm]
625 mA, 2.5 min	648	227
750 mA, 2.5 min	683	241
875 mA. 2.5 min	750	264

Whilst all the samples produced channels that could be used in further studies due to their appropriate depth and suitable width, the channel formed at 625 mA and 2.5 minutes demonstrated the lowest degree of under etching (ca. 100μm) and was also closest to the desired depth. As a result, this combination of parameters was taken as being the most suitable for the etching of UAM parts containing optical fibres.

Initially CNC machining had been trialled as a means of creating an aperture between embedded optical fibres in order to spectroscopically sample the passing solution, but was demonstrated

to be unsuitable due to irreparable damage occurring to the fibre. With no ability to pre-align the optical fibres parallel to a fluidic channel, it was decided that a more suitable method would consist of first embedding the optical fibre, then forming a fluidic channel around this structure and finally fibre cross sectioning and encapsulation of the whole structure. Initially more traditional methods of masking and etching substrates involving the application of photoresists, photomasks and chemical etchants were applied to the UAM samples. However due to the rough nature of the substrates, the generation of uniform photoresist layers with suitable levels of adhesion was a significant issue. Furthermore, the etching of aluminium using common etchants such as ferric chloride and hydrofluoric acid is hard to control and potentially dangerous. As a result, a novel process for channel formation in which the fibre is left undamaged and needs no post-treatment needed to be established to allow for potential spectroscopic analysis.

Electrochemical etching (ECE) was implemented as a means of removing excess matrix material using a non-aggressive substrate removal process. In a process similar to electroplating, ECE uses electrolytic solutions and electrical currents to remove predefined regions of material from the surface of a metallic substrate. As a result, experimentation was performed on a number of UAM substrates in order to determine the suitability of ECE as a means of manufacturing channels of suitable resolution. Following this, FIB milling was used to create an aperture between embedded fibres and their optical performance tested via spectroscopic analysis. This technology was then employed to manufacture a UV-vis flow cell.

An electrochemical etching cell was constructed using a Thurlby Thandar PL154 Direct Current (dc) power supply (Thurlby Thander, Huntingdon, UK), specially designed electrode and sample holder (formed via FDM), a glass-etching tank and an electrolytic solution of sodium

chloride. Unlike traditional etching methods, these systems require no harsh chemicals or expensive equipment. Additional information on this cell and images of this etching rig are located within the appendix.

Once the cell was manufactured, a Synrad CO_2 laser-marking system was employed (Synrad Inc., Mukilteo, United States) to form channel layouts onto the UAM in channel sizes more commonly found in flow reactor systems (< 1mm). These CO_2 marking systems are often used in order to achieve high-resolution markings on the surface of various polymer substrates. Due to the highly reflective nature of the aluminium substrate along with its high thermal conductivity, the CO_2 laser has no effect on the metallic substrate at the powers used in this work (10-20 W). The laser system was used to thermally remove channel structures into the adhesive polymer layer placed onto the surface of the substrates so that the exposed section was open to etching by the ECE cell.

A design of experiments (DOE) approach was undertaken in order to establish the relationship between channel formation and the various parameters associated with these electrolytic cells. Firstly, a preliminary trial was performed to investigate the effects of different cell parameters on the geometry of the channel formed. In order to determine the effect of the conditions within the cell on the dimensions of the fluidic channel formed; a 2-factorial, 3-level, response surface methodology (RSM) DOE was undertaken. The factors chosen for investigation were both the current of the cell (mA) as well as the total sample exposure time (min.). The levels of these different factors are located in Table 3.

Table C. DOE values used in etching initial etching investigation

Factor	Low	Centre	High
Exposure time [min.]	5	10	15
Current [mA]	500	750	1000

The channels producing the most consistent etching of appropriate dimensions (100- 500μm width and a depth > 200μm so that the entire fibre is exposed for later works in which the fibre will be cut) were then further optimised in order to produce a channel suitable for UV-vis spectroscopy. This was achieved through further design space optimisation by centering on the most suitable combination of trial parameters. Again, a 3 level, 2 factor RSM DOE was employed for this testing. The ability to etch around embedded fibres was then established by applying the optimised parameters to fibre-embedded samples. Experimental randomisation was used in order to balance the effect of extraneous or uncontrollable conditions that could have affected the results of the experiment and potentially bias results and additional centre points included in order to minimise the risk of missing non-linear relationships in the middle of the intervals.

Response measurements (both the width and depth of the channels formed) were collected through the use of an Alicona InfiniteFocus IFMG4F surface profiling system (Alicona Imaging GmbH, Grambach, Austria) using a x5 objective lens. The InfiniteFocus system used non-contact focus variation microscopy to obtain high-resolution images. The acquired data was processed using Talymap Platinum 5.0 (Taylor Hobson Ltd, UK). Six measurements were made for each substrate at random regions along the channel in order to gain an average value for both the width and depth of said channel. All measurements were taken at a temperature of 21

$\underline{+}$ 2 ^{O}C and samples were thermally conditioned at this temperature for a minimum of 24 hours prior to measurement.

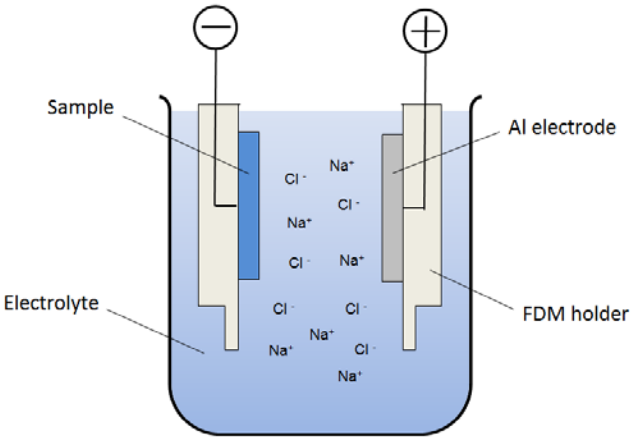

Fig C. Diagram of the electrochemical etching cell used in the etching of UAM samples

The dc power supply was chosen for its ability to provide built-in voltage control and current limiting, whilst the electrolyte used in this work was an aqueous salt solution formed from sodium chloride (concentration 200 g L^{-1}), all of which was reagent, ≥99.0. The sodium chloride solution was chosen due to its relative safety and availability. The conductivity of the cell is proportional to the concentration of the electrolyte used (approximately 2/3rd of the saturation point was used in this work). The reason for the concentration of the solution chosen here was to ensure a suitable resistance existed within the cell to not draw too much power from the external power supply, allowing for an easily controllable current and voltage. A diagram of this cell and specially designed sample and electrode holders are located in Fig C and the final rig displayed in Fig D.

This cell design ensured a constant anode-cathode separation, as well as negating the requirement to protect metal connections at both the anode and cathode, as these are also susceptible to etching when exposed to the cell. The anode was formed from Al 1050 due to the high degree of conductivity and corrosion resistance.

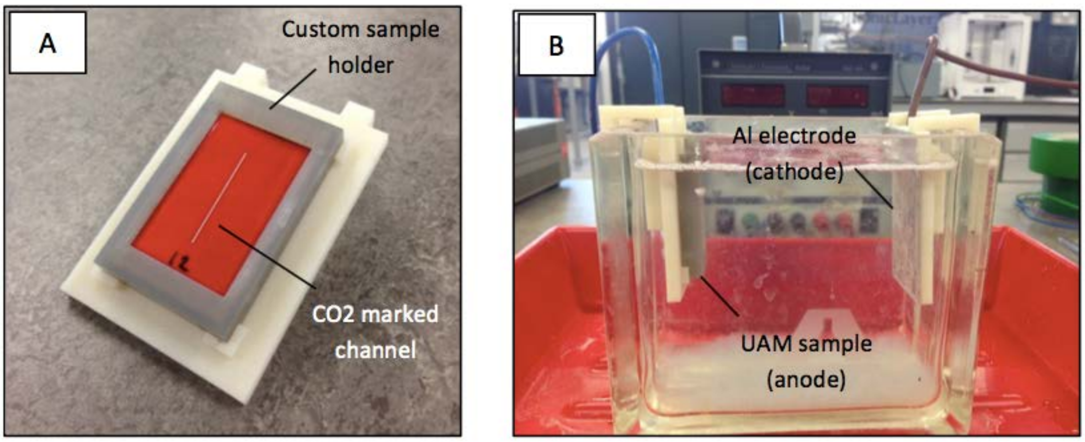

Fig D. ECE cell and components. (A) UAM sample masked with Tesa 4154 resist and marked using optimised parameters (B) sample and counter electrode configured in the ECE cell

Focused ion beam cross sectioning of embedded fibres

A novel method based upon ion beam milling and lift out procedures was then developed as a result of previous publications utilising ion beam for various experimentation regarding both UAM [3] and fibre machining [4,5].

Due to the embedded optical fibre being difficult to access with traditional tools such as scribes, an appropriate means of cross sectioning the fibre needed to be established in order for it to be useful as an optical waveguide for future spectroscopic applications. Focused ion beam (FIB) milling was previously shown by Abdi *et. al.* to be a highly suitable method in the cleaving of

polymer optical fibres (POF) in the development of strain sensors [6,7]. FIB machining has also been used in a number of other fibre-based applications, including; modification of waveguide properties [8], fabrication of long period gratings [9], micromachining of the fibre end tip for Fabry-Perot cavities in sensing applications [10], precision cutting of photonic crystal fibres [11], formation of optical lenses for efficient fibre-to-waveguide coupling [5], and the writing of gratings in silicon optical waveguides [12].

FIB offers several important advantages such as; precise site selection, milling control, and high quality surface finish. These are all properties that are highly desirable when designing a system for spectroscopic interrogation inside fluidic channels. In light of this, this section presents a novel technique for the cross sectioning of optical fibres embedded within UAM metal matrices via DBFIB technology.

A Nova 600 NanoLab Dual Beam system equipped with an SEM and uses a liquid gallium ion source was then employed for both cross sectioning and imaging of embedded fibres. This apparatus delivers a focused Ga^{+} ion beam with energy 5–30 keV and a probe current of 1 pA - 20 nA. The FIB technique started with coating the exposed fibre sample embedded with approximately 100 nm of palladium-gold using a Quorum Q150T ES sputter coater (Quorum Technologies, Lewes, UK) (Fig D - A). Cross- sectioning of the exposed fibre was comprised of two major stages: firstly a mass removal stage in which 240 μm long cross sections were milled in order to remove a large central fibre section, followed by cross-section polishing of the newly exposed surfaces in order to produce flat clean edges and ensure the aperture distance of 250 μm. The ion beam for both the ion milling and imaging was set at an accelerating voltage of 10 kV and a 10 × 20 μm^2 area removed, in stages, in the mass removal step using a 20 nA aperture and a rectangular milling box.

In the mass removal stage, sections were removed from edge to edge of the fibre as opposed to performing one large single cut (Fig E - B). This was due to the edges of the fibre tending to mill at a faster rate than the central regions due to re-deposition of milled material back onto the bulk, whereas material at the edges typically does not redeposit in the local area as there is less surface area for it to redeposit on. A small amount of material was left un-milled in the second cut in order to keep this cut section connected to the bulk of the fibre (Fig E - C). The coaxial OmniProbe nano-manipulator integrated into this DBFIB system was then used to manipulate this fibre section and detach it from the bulk. These probes are ideal for nanoscale positioning and manipulations and are commonly called upon to execute FIB lift-out procedures.

A 5 nA aperture was used for cross-section polishing of fibre faces, with an assisted cleaning cross-section milling box providing a line-by-line scan. The initial distance between the two sets of cuts was set at 240 μm, with an additional 10 μm being removed in cross-section polishing. This distance was chosen in order to form a balance between the concentrations of analytes/reagents it is possible to monitor in the device. This is based upon standard practice within UV-vis spectroscopy whereby smaller distances typically excel for low concentration monitoring whilst larger distances are more suitable for higher concentrations. Imaging was then performed using an ion beam with a 100 pA aperture and SEM. An ion beam was chosen to perform the imaging due to the lack of secondary electron signal generated by the sample because of the insulating nature of the cross- section surface.

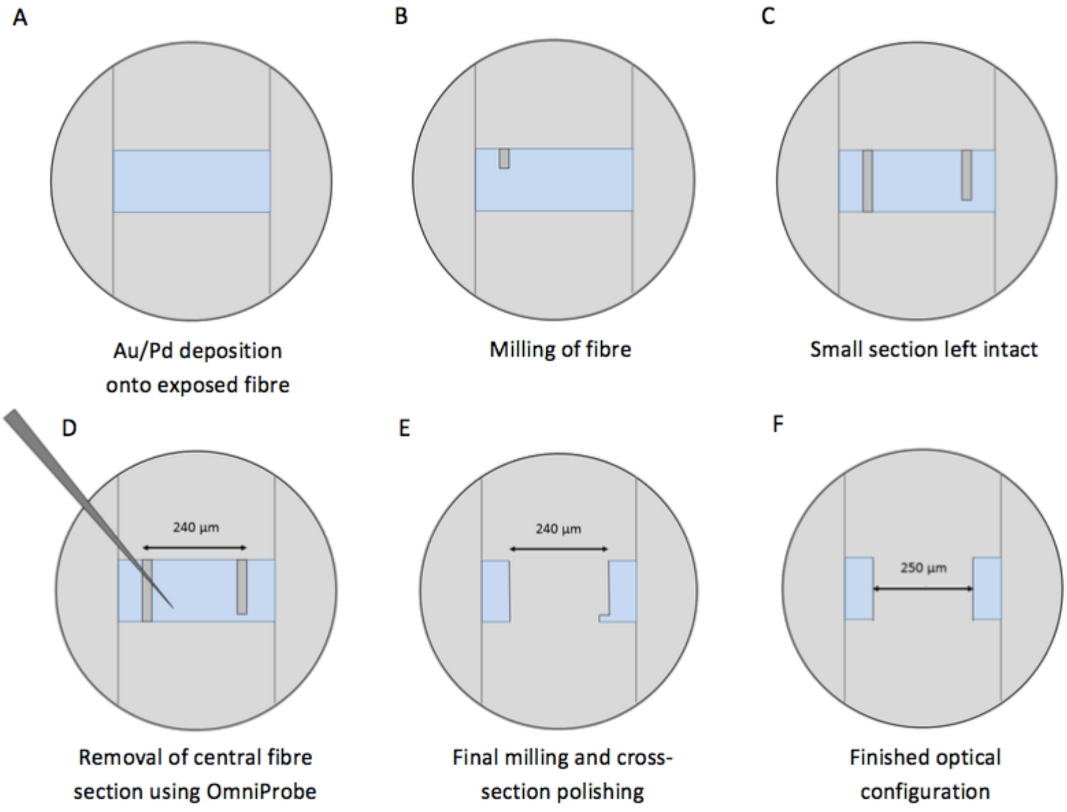

Fig E. Stages of FIB milling of embedded optical fibre (blue) (A) deposition of Au/Pd onto fibre (B) milling of fibre from edge to edge (C) leave small amount of fibre remaining at one end (D) welding of the coaxial OmniProbe nanomanipulator to optical fibre segment (E) removal of optical fibre segment (F) finalised milling and polishing of fibre faces

Focused ion beam milling of embedded optical fibres

Using ion beam milling, a method of cross-sectioning embedded optical fibres was developed and found to be highly suitable in the formation of optical configurations suitable for UV- vis spectroscopy. A pictorial representation of the stages is located in Fig F – Fig I.

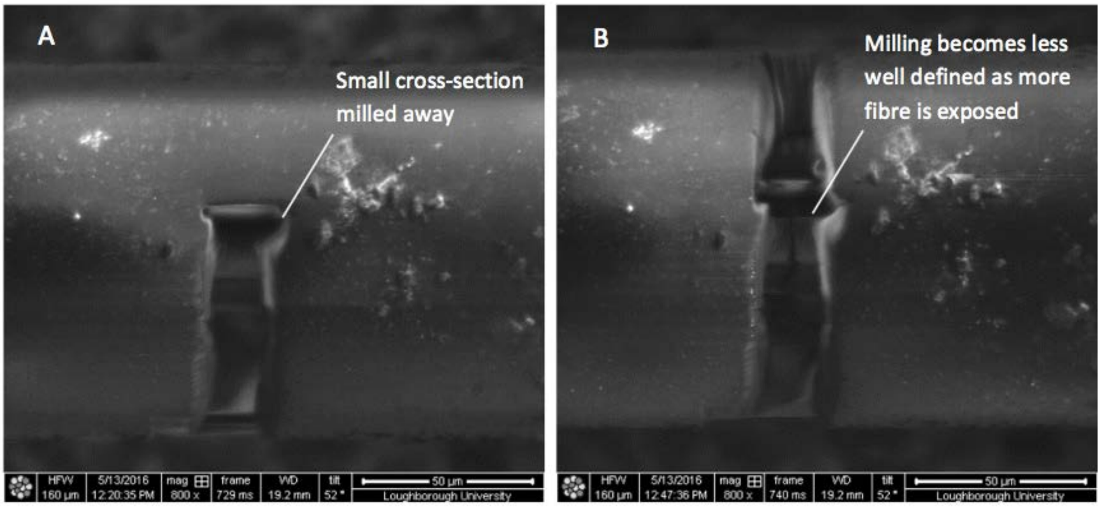

Fig F. (A) Initial milling of fibre in small stages (B) image drift resulting in distortion of milling process

The fibre was initially cross-section using the ion beam to mill away small rectangular segments, from one side of the fibre to the other (Fig F). Due to small degrees of charging of the substrate because of the non-conductive nature of the newly exposed fibre, drifting of the image was noted during milling. This resulted in a slightly more distorted cut that had initially been planned. Due to issues with the highly charged surfaces occasionally causing milled away sections to stick to the remaining bulk material, a small section of the fibre was left intact in order to allow manual removal of this cross-section fragment.

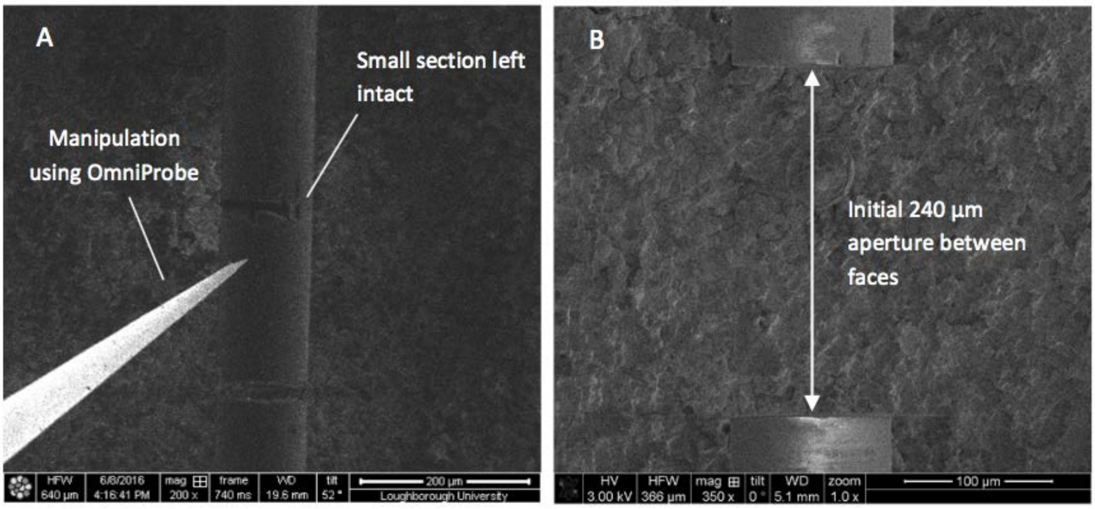

Fig G. (A) Removing of fibre segment using the OmniProbe to create (B) aperture between fibres

Employing the DBFIB coaxial OmniProbe needle to manipulate this cross-section segment, the segment was removed to create an aperture of 240 µm between the two new faces of the fibre (Fig G). The faces of these fibres were coarse and required additional polishing in order to produce both clean flat faces and adjust the separation distance to the desired 250 µm (Fig G).

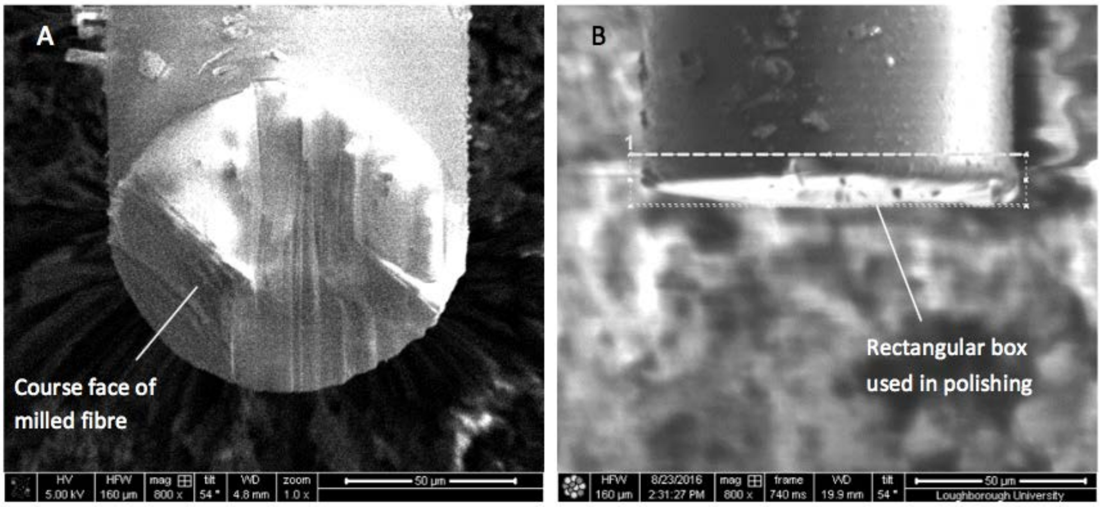

Fig H. (A) Course unpolished face of milled fibre (B) Rectangular milling box used in cross-section polishing

Using a reduced aperture, these faces were highly polished to yield clean, flat surfaces that are highly desirable for the transmission and receiving of light across a band gap (Fig I). In spectroscopy, this reduces coupling losses and could potentially yield increases in sensitivity.

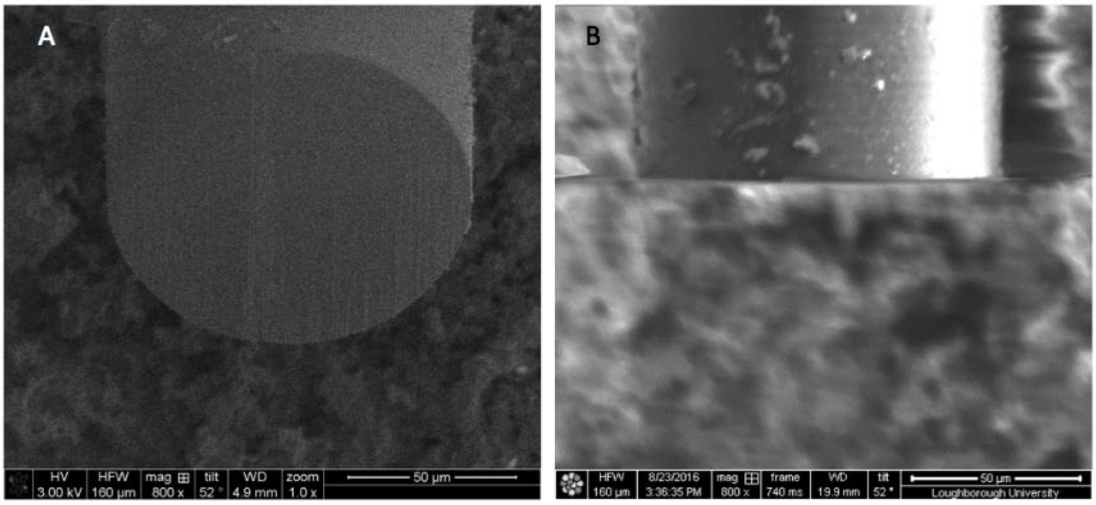

Fig I. (A)-(B) Clean faces produced through cross-section polishing highly suitable for spectroscopic applications

Concluding this final polishing stage, the etching channel and fibre were encapsulated with a covering Al 3003 foil layer at the same parameters and previously used for substrate manufacture. The total time to cut through the fibre and polish both faces of the fibre was approximately eight hours. Once embedded, the system was taken forward for spectroscopic testing in regards to the transmission performance of the cross-sectioned fibres.

Stages of manufacture in the development of UAM UV-vis cell

Stage 1

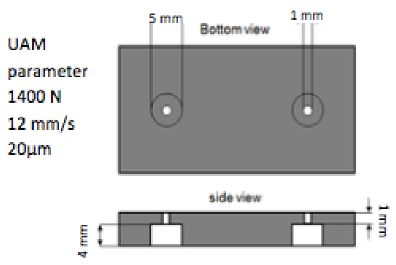

Initially a UAM substrate was prepared via deposition of three individual Al 3003 foils onto a base plate at parameters described in chapter 5 (1400 N, 12 mm s^{-1}, 20 µm). A single Inlet and outlet were then machined into the underside of the baseplate in order to allow for fluidic fittings to be attached at a later stage for testing.

Stage 2

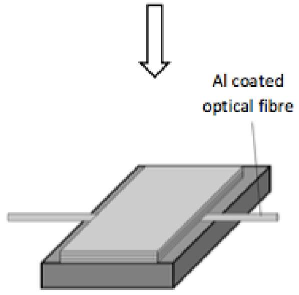

Concluding this machining, an aluminium coated optical fibre was embedded between the top surface of the substrate and a fourth Al 3003 foil at the same welding parameters. In accordance with previous findings (Chapter 6) the fibres were embedded so that the sonotrode traverse direction was along the length of the fibre in order to reduce trauma to the glass core and allow for the highest possible light transmission.

Stage 3

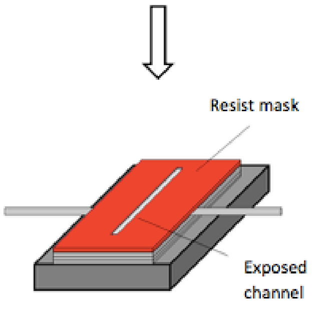

Once embedded, the top surface of the substrate was masked with a resistive layer and processed so that a small surface channel was removed, allowing the underlying substrate to be accessible to an etching solution. The methods behind the application and further processing of this layer are located in Section 7.3.2. The substrate was then etched, the masking layer removed and the fibre cross-sectioned using the DBFIB methodology previously described.

Stage 4

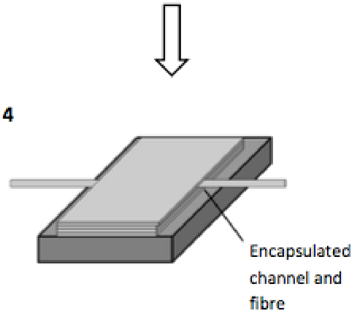

Once etched and cross-sectioned, a final Al 3003 covering layer was applied to the top surface of the substrate, encapsulating the fibre and channel. The cell was then equipped with the appropriate fluidic fittings (1/4" 28 UNF) and taken forward for spectroscopic testing and analytical measurements.

Fig J. Process chain of UV-vis UAM reactor cell manufacture (Stage 1) preliminary machining (Stage 2) fibre embedding (Stage 3) masking, etching and cross-sectioning (Stage 4) encapsulation of channel and fibre

Material Properties of Metallic Foils

Table D. Mechanical properties and chemical composition of Al 1050 H14 and Al 3003 H18

Material property	Al 1050 H14	Al 3003 H18
Density [g/cm^3]	2.71	2.73
UTS [MPa]	100-135	200
Tensile Yield Strength [MPa]	75	186
Modulus of Elasticity [GPa]	69	68.9
Melting Temperature [°C]	645-657	643-654
Composition [wt%]	Al (≥99.08), Mn (≤0.05), Cu (≤0.05), Fe (≤0.4), Si (≤0.25), Zn (≤0.07), Mg (≤0.05), Ti (≤0.05), Other (≤0.03)	Al (96.7–99), Mn (1–1.5), Cu (0.05–0.2), Fe (<0.7), Si (<0.6), Zn (<0.1) Other (<0.15)

Table E. Dimensions and coating compositions of metallised optical fibres

	Al 100-110
Primary coating	Aluminium (99.99% purity)
Core diameter [μm]	100 ± 2
Cladding diameter [μm]	110 ± 1
Coating diameter [μm]	200 ± 10

Preliminarily etching trials response contour plots

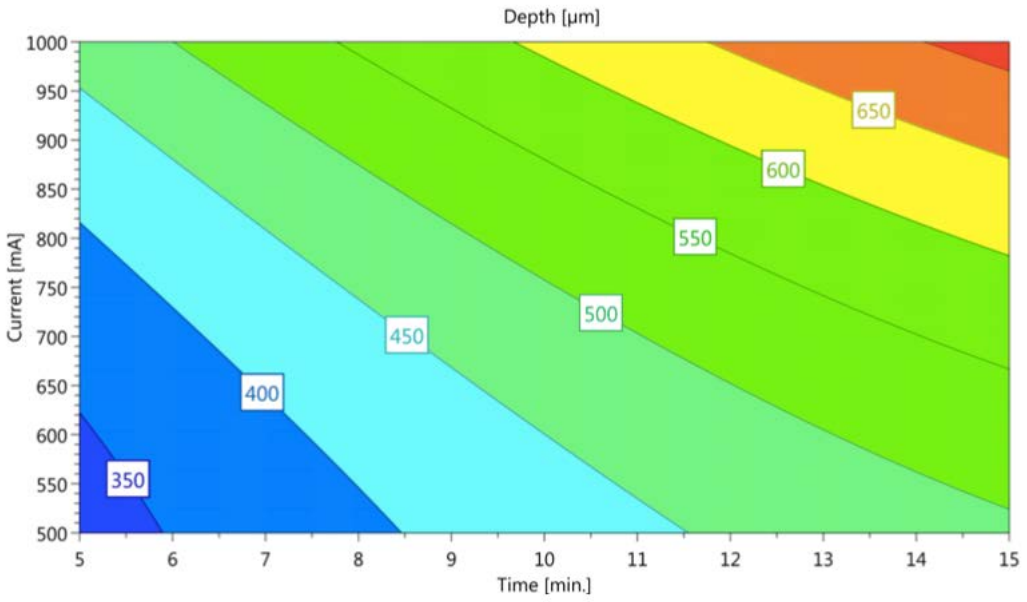

Fig K. Response contour plot showing the change in channel depth with respect to current and time

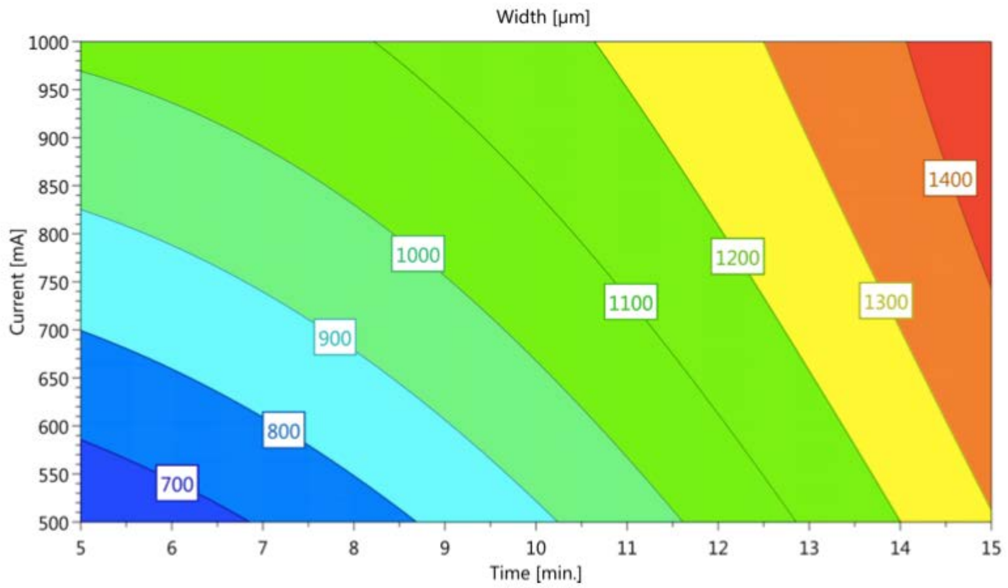

Fig L. Response contour plot showing the change in channel width with respect to current and time

Optimised etching trials response contour plots

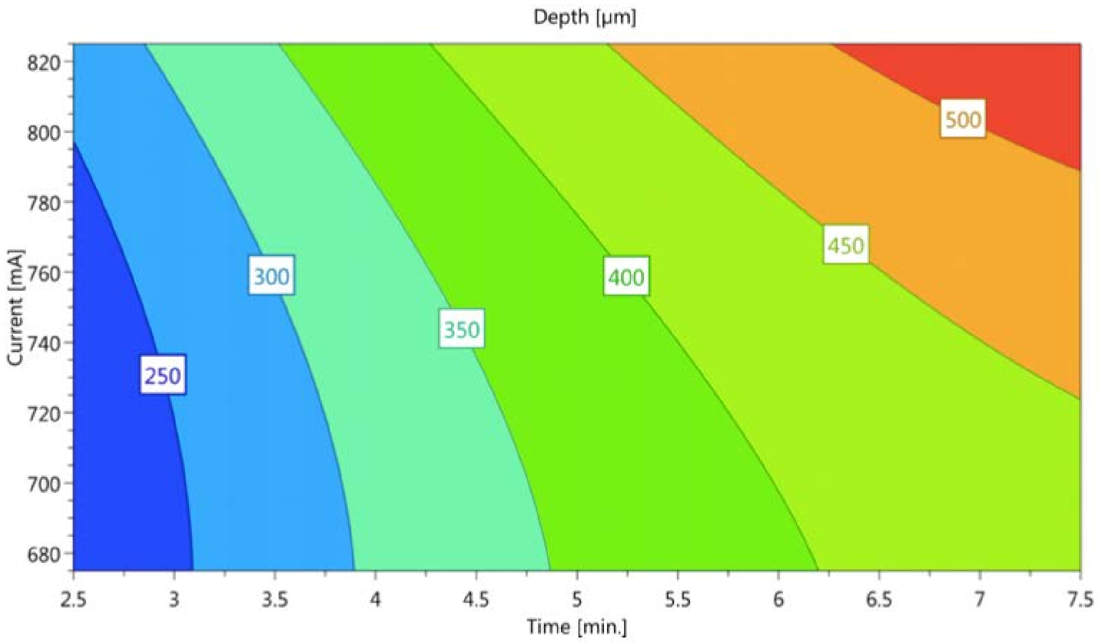

Fig M. Response contour plot showing the change in channel depth with respect to current and time

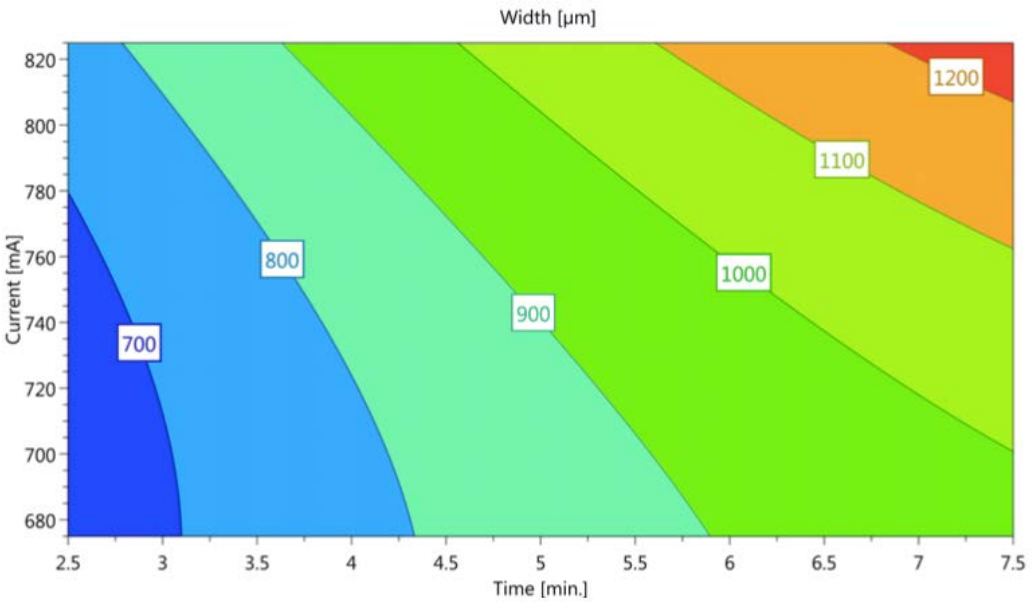

Fig N. Response contour plot showing the change in channel width with respect to current and time

Table F. Absorbance values for nicotinamine solutions in UAM cell

Concentration	Absorbance @ 260 nm
1.00	0.042
2.50	0.101
5.00	0.260
7.50	0.416
10.0	0.531
12.5	0.701
15.0	0.815
20.0	1.125
25.0	1.388
30.0	1.641
**R^2**	0.999
**Intercept**	0.018

Table G. Absorbance values for fluorescein solutions in UAM cell

Concentration	Absorbance @ 494 nm
0.010	0.000
0.020	0.003
0.041	0.019
0.081	0.070
0.163	0.156
0.325	0.314
0.750	0.795
1.000	1.055
1.500	1.591
**R^2**	0.999
**Intercept**	0.020

Manufacture and testing of UAM UV-Vis flow system

Combining all of the methods described in this work, the first reported case of a UAM UV-Vis flow cell was manufactured.

UAM processed Al 3003-H18 substrates were first manufactured at process parameters proven conducive to achieving high degrees of mechanical performance whilst still allowing functionality of embedded optical fibres. These substrates were then exposed to an electrochemical etching cell in order to form channels with dimensions similar to that commonly employed in flow chemistry. This allowed for an evaluation of how the main variables of the cell affected the dimensions of the newly formed channel. This was achieved through a combination of DOE, non-contact focus variation microscopy and spectroscopic measurements.

This research has outlined how UAM can be used to develop the flow reactors featuring discretely embedded sensors within specifically selected materials whilst maintaining the design freedom associated with additive manufacturing. These advantages could enable a much wider range of reactions and experimentation to be investigated and is more likely to be applicable to modern research and industrial chemistry due to their robust nature lower cost. This can potentially move away from the concept of a reactor chip in a lab and move toward the desired goal of a lab-on-a-chip.

References

1. Monaghan T, Capel AJ, Christie SD, Harris RA, Friel RJ. Solid-state additive manufacturing for metallized optical fiber integration. Compos Part A Appl Sci Manuf. 2015;76. doi:10.1016/j.compositesa.2015.05.032
2. Monaghan T, Harding MJ, Harris RA, Friel RJ, Christie SDR. Customisable 3D printed microfluidics for integrated analysis and optimisation. Lab a Chip - Miniaturisation Chem Biol. 2016;16: 3362–3373. doi:10.1039/c6lc00562d
3. Dehoff RR, Babu SS. Characterization of interfacial microstructures in 3003 aluminum alloy blocks fabricated by ultrasonic additive manufacturing. Acta Mater. 2010;58: 4305–4315. doi:10.1016/j.actamat.2010.03.006
4. Martelli C, Olivero P, Canning J, Groothoff N, Gibson B, Huntington S. Micromachining structured optical fibers using focused ion beam milling. Opt Lett. 2007;32: 1575. doi:10.1364/OL.32.001575
5. Schiappelli F, Kumar R, Prasciolu M, Cojoc D, Cabrini S, De Vittorio M, et al. Efficient fiber-to-waveguide coupling by a lens on the end of the optical fiber fabricated by focused ion beam milling. Microelectron Eng. 2004;73: 397–404. doi:10.1016/j.mee.2004.02.077
6. Abdi O, Wong KC, Hassan T, Peters KJ, Kowalsky MJ. Cleaving of solid single mode polymer optical fiber for strain sensor applications. Opt Commun. 2009;282: 856–861. doi:10.1016/j.optcom.2008.11.046
7. Peters K. Polymer optical fiber sensors—a review. Smart Mater Struct. 2011;20: 013002. doi:10.1088/0964-1726/20/1/013002
8. von Bibra ML, Canning J, Roberts A. Mode profile modification of H+ ion beam irradiated waveguides using UV processing. J Non Cryst Solids. 1998;239: 121–125. doi:10.1016/S0022-3093(98)00727-3

9. von Bibra ML, Roberts A, Canning J. Fabrication of long-period fiber gratings by use of focused ion-beam irradiation. Opt Lett. 2001;26: 765. doi:10.1364/OL.26.000765

10. André RM, Pevec S, Becker M, Dellith J, Rothhardt M, Marques MB, et al. Focused ion beam post-processing of optical fiber Fabry-Perot cavities for sensing applications. Opt Express. 2014;22: 13102. doi:10.1364/OE.22.013102

11. Gibson BC, Huntington ST, Rubanov S, Olivero P, Digweed-Lyytikäinen K, Canning J, et al. Exposure and characterization of nano-structured hole arrays in tapered photonic crystal fibers using a combined FIB/SEM technique. Opt Express. 2005;13: 9023. doi:10.1364/OPEX.13.009023

12. Moss DJ, Ta'eed VG, Eggleton BJ, Freeman D, Madden S, Samoc M, et al. Bragg gratings in silicon-on-insulator waveguides by focused ion beam milling. Appl Phys Lett. 2004;85: 4860. doi:10.1063/1.1824182
